# Gene profiling reveals the role of inflammation, abnormal uterine muscle contraction and vascularity in recurrent implantation failure

**DOI:** 10.3389/fgene.2023.1108805

**Published:** 2023-02-24

**Authors:** Xinyi Dong, Mi Zhou, Xinyu Li, Huijing Huang, Yun Sun

**Affiliations:** ^1^ Center for Reproductive Medicine, Renji Hospital, School of Medicine, Shanghai Jiao Tong University, Shanghai, China; ^2^ Shanghai Key Laboratory for Assisted Reproduction and Reproductive Genetics, Shanghai, China; ^3^ Department of Rheumatology, Renji Hospital, School of Medicine, Shanghai Jiaotong University, Shanghai, China; ^4^ Department of Rheumatology, Zhongshan Hospital, Fudan University, Shanghai, China

**Keywords:** recurrent implantation failure, inflammation, contraction, vascularity, expression profiling

## Abstract

**Objective:** Recurrent implantation failure (RIF) is now disturbing numerous infertile couples accepting assisted reproductive technology (ART). And the endometrial factors are crucial causes of recurrent implantation failure. However, its mechanism is still unclear. Thus, the aim of this study is to identify altered biologic processes in endometrium that may contribute to recurrent implantation failure.

**Methods:** We recruited two microarray datasets (GSE103465, GSE111974) from Gene Expression Omnibus database (GEO), which contain endometrium from RIF and normal women during implantation period. Using the online tools GEO2R and Venny, we identified Differentially Expressed Genes (DEGs) of selected datasets, and obtained common DEGs. Gene Ontology (GO) terms, Kyoto Encyclopedia of Genes and Genomes (KEGG) and BioCatar pathway enrichment were conducted with Enrichr platform, “ssgsea” and “ggplot2” package of RStudio. PPI networks and hub gene related TF-gene interaction and TF-miRNA co-regulation networks were built *via* online tools STRING and NetworkAnalyst. Immune infiltration analysis was performed by CIBERSORT platform. Recurrent implantation failure subgroup identification was achieved through “ConsensusClusterPlus,” “tsne,” “ssgsea”, and “ggpubr” package in RStudio. Diagnostic characteristic ROC curves were constructed *via* “pROC” and “ggplot2” package of RStudio. Enrichr platform was utilized to find drugs targeting hub genes.

**Results:** 26 common DEGs were confirmed. Gene Ontology and Kyoto Encyclopedia of Genes and Genomes/BioCarta analysis determined common DEGs were mainly enriched in inflammation associated pathways including TNF, NF-κB, IL-4, IL-10, IL-6, and TGF-β signaling pathways. Five hub genes (*PTGS2*, *VCAM1*, *EDNRB*, *ACTA2*, and *LIF*) and related TF-gene and TF-miRNA interactions were identified. Immune infiltration analysis indicated the importance of macrophage M2 in recurrent implantation failure patients. Importantly, subgroup identification analysis highlighted that recurrent implantation failure patients can be divided into two subgroups with different phenotypes. Moreover, the ROC curves and drugs may provide new diagnostic and therapeutic thought for recurrent implantation failure.

## Introduction

Nowadays, infertility depresses 8–12% of couples in reproductive age worldwide, and the boom of assisted reproductive technology (ART) has allowed numerous infertile couples to achieve feasible pregnancy (INHORN AND PATRIZIO, 2015). However, a challenging problem arising in this domain is recurrent implantation failure (RIF) (Bashiri et al., 2018) As far as we know, there is still lacking a world-wide acknowledged formal definition of RIF, but a relatively recognized definition is that RIF is failure in three *in vitro* fertilization-embryo transfer (IVF-ET) cycles after transferring good quality embryos (Orvieto et al., 2015; Bashiri et al., 2018). Among patients under infertility treatment, 15% suffer from RIF (Busnelli et al., 2020; Mrozikiewicz et al., 2021).

Implantation is a complex process requiring precise embryo-uterine cross-talk, which is still not well understood (Mrozikiewicz et al., 2021). The window of implantation (WOI) is a strict time span when blastocyst is overlain on the receptive state of the endometrium. Abnormality of each link in implantation can lead to RIF (Mrozikiewicz et al., 2021).

Risk factors of RIF include maternal age, smoking, stress and so on (Orvieto et al., 2015; Bashiri et al., 2018). Immunological factors including peripheral and uterine natural killer cells, Th1/Th2 ratio, tumor necrosis factor alpha (TNF-α) levels, auto-antibodies, antiphospholipid syndrome, hereditary thrombophilia as well as infection are considered to participated in the pathogenesis of RIF (Bashiri et al., 2018). Endometrium is the place of embryo to locate, adhere, penetrate and develop in. Abnormal status of endometrium, such as chronic endometritis, embryo-endometrial asynchrony, endometrial injuries (e.g., pipelle catheter, hysteroscopy and saline infusion) are factors to explain the origin of RIF (Bellver and Simón, 2018). Previous studies have reported that two-thirds of the RIF are caused by the abnormality of endometrial receptivity, so it is of great importance to focus on the role of endometrium in RIF (Margalioth et al., 2006). Various therapies are now being explored to treat RIF including different types and methods of embryo transfer, ovulation induction protocol, progesterone support, antithrombotic agents, immunotherapy, anti-infection, anatomical intervention and so on (Bashiri et al., 2018). However, the problem isn’t fully resolved by the above-mentioned therapies, and we still dont have an ideal method to detect the causes of every RIF individual. Therefore, it is urgent to conduct bioinformatic analysis aiming to find potential mechanism and effective treatment of RIF.

**FIGURE 1:**
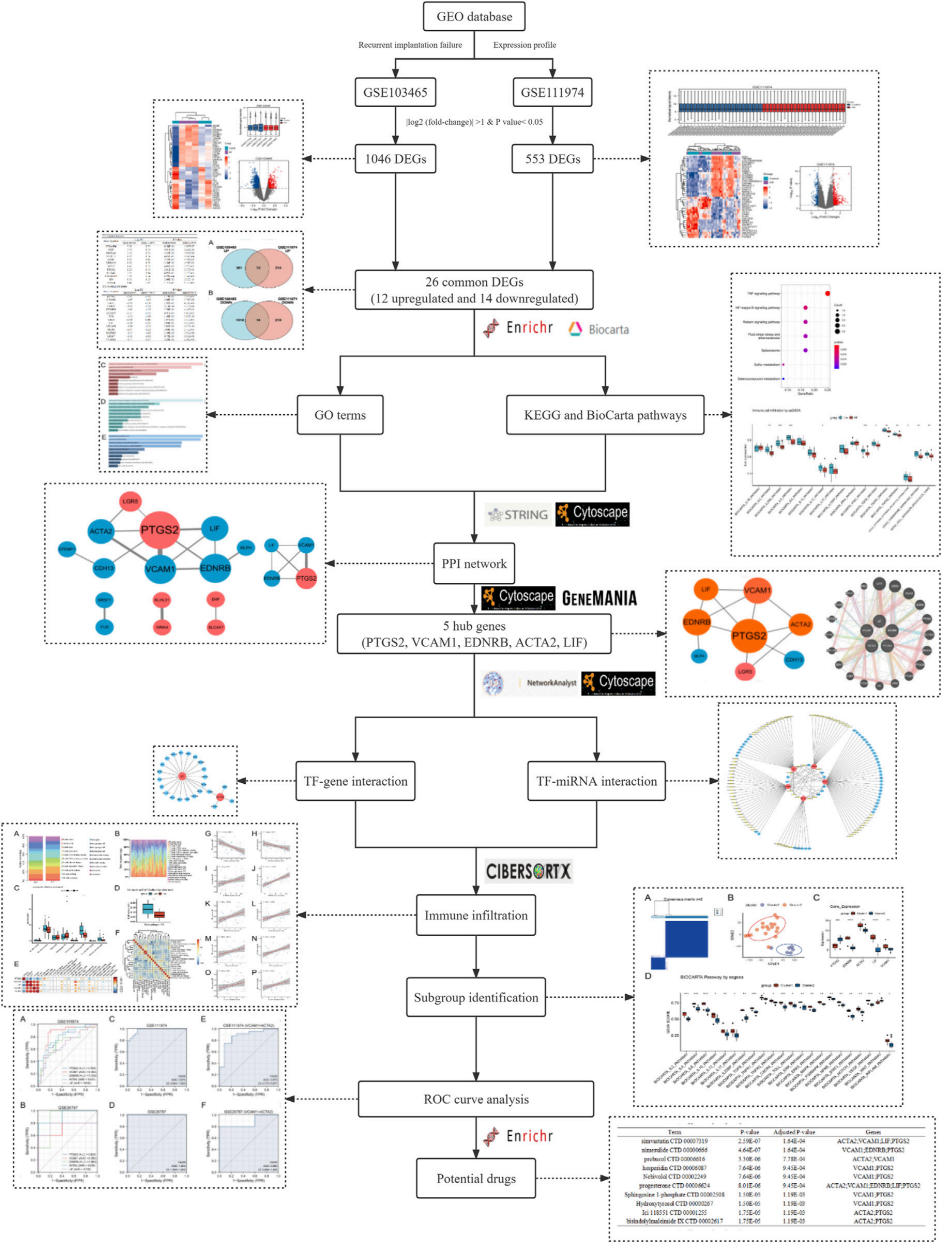
Methodical workflow for the current investigation.

Accumulating evidence has demonstrated that many genes have been proposed as potential receptivity markers, however, considering heterogeneity among those independent experiments as an outcome of variations in specimens or tissue and different data processing methods, the identification of those Differentially Expressed Genes (DEGs) is inconsistent. Therefore, in this research, we attempt to find more effective and reliable biological pathways and potential biomarkers contributing to the pathogenesis and development of RIF *via* integrating different studies. We downloaded two microarray datasets GSE103465 and GSE111974 from Gene Expression Omnibus database (GEO), which contain gene expression profiling from endometrial tissues of women with RIF and fertile women during WOI. We then performed further bioinformatic analysis, including common DEGs identification, gene ontology (GO), Kyoto Encyclopedia of Genes and Genomes (KEGG)/BioCarta pathway enrichment, protein-protein interaction (PPI) network analysis, TF-genes and TF-miRNA interaction analysis, subgroup identification, immune infiltration analysis, characteristic (ROC) curve analysis and drug searching The workflow of our analysis is displayed in Figure 1.

## Materials and methods

### Original data collection

We input “recurrent implantation failure” and “expression profile” as two keywords to the GEO database, then two datasets GSE103465 and GSE111974 were selected for analysis, and GSE26787 was chosen for validation. Both GSE103465 and GSE111974 contain expression profiles of endometrial tissue obtained from RIF and control women during WOI. GSE103465, in GPL16043 platform, contains whole-genome expression profiles of endometrial tissue from three women divided to the control group and RIF group (Guo et al., 2018). In GSE103465, RIF is defined as no pregnancy after ≥3 embryo transfers including a total of ≥4 good-quality embryos, and inclusion criteria of control group is infertile women with tubal factors who achieved a clinical pregnancy after the first embryo transfer (Guo et al., 2018). GSE111974, in GPL17077 platform, consists of 24 individuals with RIF and 24 fertile control patients, in which RIF is determined as failure of pregnancy in three consecutive IVF cycles with at least one transfer of good quality embryo in each cycle, while the fertile control refers to patients who had a history of at least one live birth with no related comorbidities (Bastu et al., 2019). The platform and series matrix files were all downloaded.

### Analysis for DEGs

Using the online analysis tool GEO2R (https://www.ncbi.nlm.nih.gov/geo/geo2r/), the expression profiles of endometrium from RIF patients and fertile women were compared to screen the DEGs of the two datasets, independently. *p* values were calculated through t-tests and genes with the criteria of a |log2 (fold-change)| >1 and *p*-value < 0.05 were considered as DEGs. The volcano plot and box diagram were both created *via* the “ggplot2” package of RStudio software, and the heatmap for the DEGs was drawn using the “ComplexHeatmap” package of RStudio software (Gu et al., 2016; Wickham, 2016). Overlapping DEGs from two databases were defined as common DEGs and were displayed with Venn diagrams, which was drawn by utilizing the online platform Venny 2.1 (http://bioinfogp.cnb.csic.es/tools/venny/index.html).

### Gene set enrichment analysis for GO terms, KEGG and BIOCARTA pathway finding

The GO terms of common DEGs were conducted with online tool Enrichr (https://amp.pharm.mssm.edu/Enrichr/). Significantly enriched function annotations were defined as GO terms and KEGG pathways with *p* values of <0.05 (Zhou et al., 2019). The GO analysis, including biological process (BP), cellular component (CC) and molecular function (MF), provides a set of concepts for describing molecular activity and the location where the genes execute their functions (Ashburner et al., 2000). The bubble plot of KEGG pathways used to understand specific metabolic pathways of common DEGs, were visualized *via* “ggplot2” package of RStudio software (Kanehisa and Goto, 2000; Wickham, 2016). To take a further step on gene enrichment in individual samples, we performed a single sample version of gene set enrichment analysis (ssGSEA) by “GSVA” package of RStudio software, which rules an enrichment score as the degree of absolute enrichment of a gene dataset in each sample with a designated database called BioCarta (Hänzelmann et al., 2013). The differences of enrichment scores were identified *via* wilcoxon test, and were visualized by “ggpubr” package in RStudio software (Kassambara, 2020).

### Construction of PPI networks and identification of hub genes

Common DEGs are inserted into an online database called Search Tool for the Retrieval of Interacting Genes (STRING) (https://string-db.org/) to generate Protein-Protein Interaction (PPI) network. Those with a high level of confidence were regarded as valid interactions, and we set a convincing confidence score as 0.25 (Cao et al., 2021b). The obtained PPI network was then analyzed by Cytoscape 3.8.2 for a better visualization. The app Molecular Complex Detection (MCODE) on Cytoscape was applied to conduct the gene network clustering analysis to refine key modules, with a *p* < 0.05. The app Cytohubba on Cytoscape was used to compute the degrees of nodes in PPI work, proteins with high degree might have key physiological regulatory functions, so the ones having the most interactions were considered as hub genes (Cao et al., 2021a). Additionally, we used a web-based tool GeneMANIA (http://genemania.org) to further visualize the interactions and roles of hub genes.

### Analysis of TF-gene interactions and TF-miRNA co-regulation

TF-gene interactions with the identified hub genes point out the outcome of TF on functional pathways and expression levels of the genes (Ye et al., 2019). NetworkAnalyst (https://www.networkanalyst.ca/), a comprehensive online platform for analyzing gene expression, was used to identify TF-gene interaction and TF-miRNA co-regulation of identified hub genes. Then the two networks were mapped on Cytoscaope 3.8.2 for beautifying.

### Immune infiltration analysis

With the help of Cell-type Identification By Estimating Relative Subsets Of RNA Transcripts (CIBERSORT) (https://cibersort.stanford.edu/), a web-based stool able to describe cell composition of complex tissues *via* their gene expression levels, we deconvoluted and compared the cellular composition of the two groups in our analysis. Then the box plots, bar charts, heat maps and scatter diagrams revealing the association of input datasets and immune pathways and cells were all drawn through “ggpubr” packages of RStudio software (Kassambara, 2020).

### Identification of RIF subgroups

Consensus matrix of RIF subgroups identification in GSE111974 was obtained through “ConsensusClusterPlus” package of RStudio software, aiming to figure out if the five hub gene can distinguish the different subtypes of RIF(Wilkerson and Hayes, 2010). Optimal number of clusters was calculated *via* k-medoids clustering, indicating that k = 2. In order to verify the sample clustering condition of the 2 clusters we discerned, we conducted a diminished reduction analysis *via* “tsne” package of RStudio software (Donaldson, 2022). After clustering, we tried to find out the functional differences of the clusters. We used “ggpubr” package in RStudio software for visualizing the expression of hub genes in the 2 clusters, and subsequently utilized BioCarta database and “GSVA” package of RStudio software to identify the concerned pathways in the 2 clusters (Hänzelmann et al., 2013; Kassambara, 2020).

### ROC curve analysis

For the purpose of identifying the role of the five hub genes in prediction of RIF, we conducted the characteristic ROC curves of the diagnostic model in the GSE111974, *via* “pROC” and “ggplot2” package of RStudio software (Robin et al., 2011; Wickham, 2016). To validate the results, we repeated the process in GSE26787, another dataset including RIF and control group.

### Identification of potential drugs

Drug molecule identification is a pivotal component of the present study. We input five hub genes into the Drug Signatures database (DSigDB) on Enrichr (https://amp.pharm.mssm.edu/Enrichr/), thus obtained the candidate drugs interacting with hub genes, which may contribute to the treatment of RIF.

## Results

### Identification of DEGs using integrated bioinformatic analysis

**FIGURE 2:**
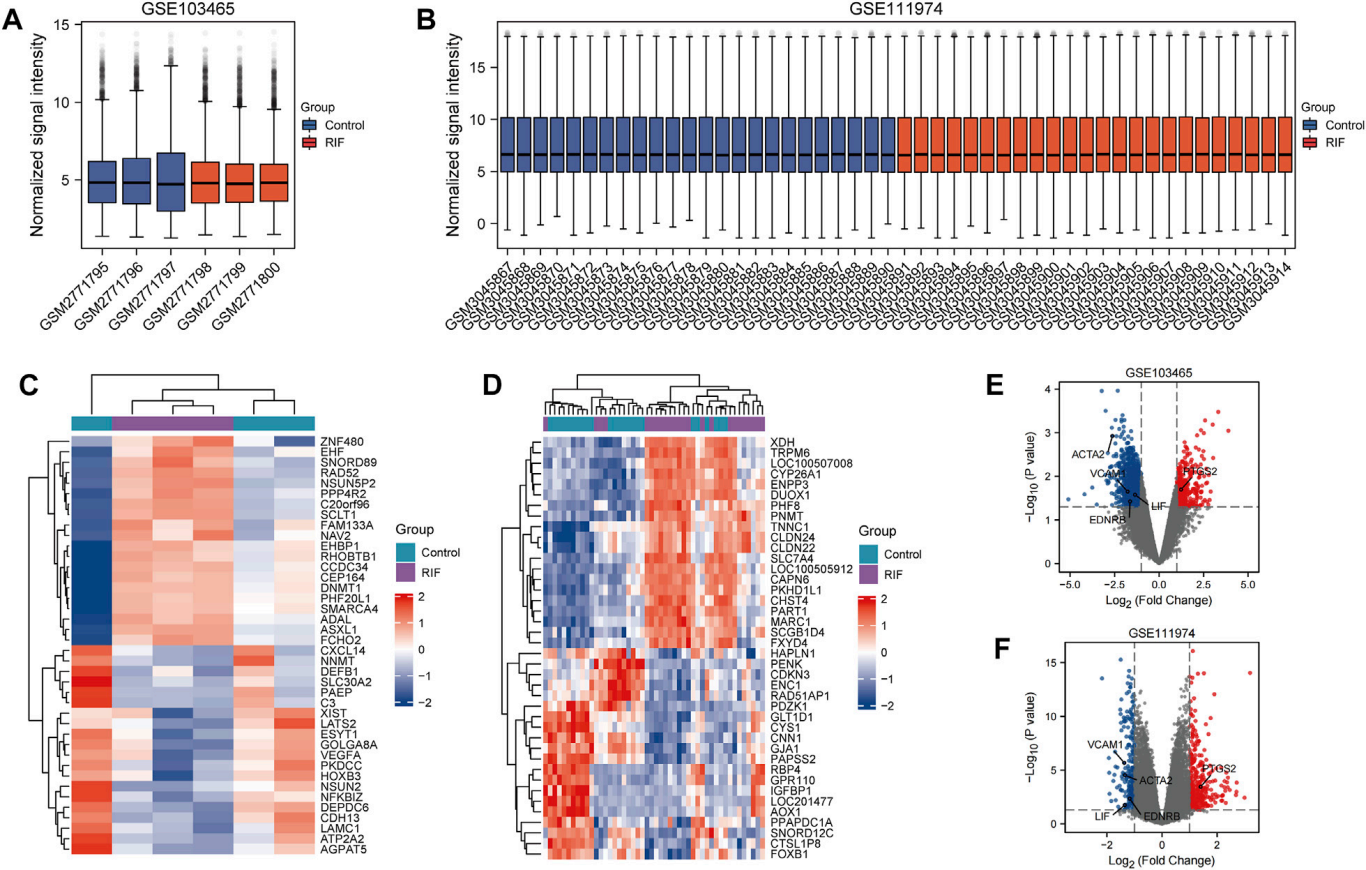
Box plots, Heatmaps and Volcano plots of DEGs between RIF and control group. **(A, B)** Box plots of GSE103465 **(A)** and GSE111974 **(B)**. Red refers to RIF group, while blue represents control group. **(C, D)** Heatmaps of 10 DEGs who have the highest log FC and 10 DEGs with the lowest log FC from GSE103465 **(C)** and GSE11194 **(D)**. Red refers to the elevated genes, and blue directs to the downregulated genes. **(E, F)** Volcano plots of DEGs in GSE103465 **(E)** and GSE111974 **(F)**. Red refers to the increased DEGs, and blue points to the reduced DEGs.

The particulars of selected three datasets were presented in Table 1, and we finally chose GSE103465 and GSE111974 for analysis. As GSE26787 is originally aimed to identify the difference of endometrium from RIF and recurrent miscarriages, and didn’t provide the specific list of individuals accepting IVF or ICSI, we used it for validation instead of analysis. 1,406 DEGs were obtained including 373 upregulated and 1,033 downregulated genes in GSE103465, while in GSE111974, 553 DEGs were collected, among which 326 genes were elevated and 227 were suppressed. The box plots shown in Figure 2 revealed the satisfying standardization of the samples. The expression of the top 20 DEGs for both two datasets were visualized on heatmaps (Figure 2). The volcano plots in Figure 2 highlighted the DEGs of the two datasets.

**TABLE 1:** Characteristics of the three GEO datasets selected.

GSE	GPL	Experiment type	Citation	Samples	Character	
103,465	16,043	Expression profiling by array	Guo et al. (2018)	Group: RIF (n = 3), fertile controls (n = 3). Sampling time: WOI (LH+7)	Women recruited in the RIF group had a history of implantation failure from at least three consecutive IVF attempts (including a total of ≥4 good-quality embryos)	The two datasets both contain the expression profile of endometrial tissue from RIF after IVF and control patients during WOI. Therefore, we chose them for analysis
111,974	17,077	Expression profiling by array	Bastu et al. (2019)	Group: RIF (n = 24), fertile controls (n = 24). Sampling time: WOI (LH+7–10)	RIF was determined as failure of pregnancy in ≥3 consecutive IVF cycles with ≥1 transfer(s) of good quality embryo in each cycle	
26,787	570	Expression profiling by array	Lédée et al. (2011)	Group: RIF (n = 5), fertile controls (n = 5), recurrent miscarriage (n = 5). Sampling time: 7–9 days after ovulation	It is originally aimed to identify the difference of endometrium from RIF and recurrent miscarriages, and did not provide the specific list of individuals accepting IVF or ICSI. Considering above mentioned reasons, we used it for the verification of ROC curves in our context	

### GO terms and KEGG/BioCarta pathway enrichment of common DEGs

**FIGURE 3:**
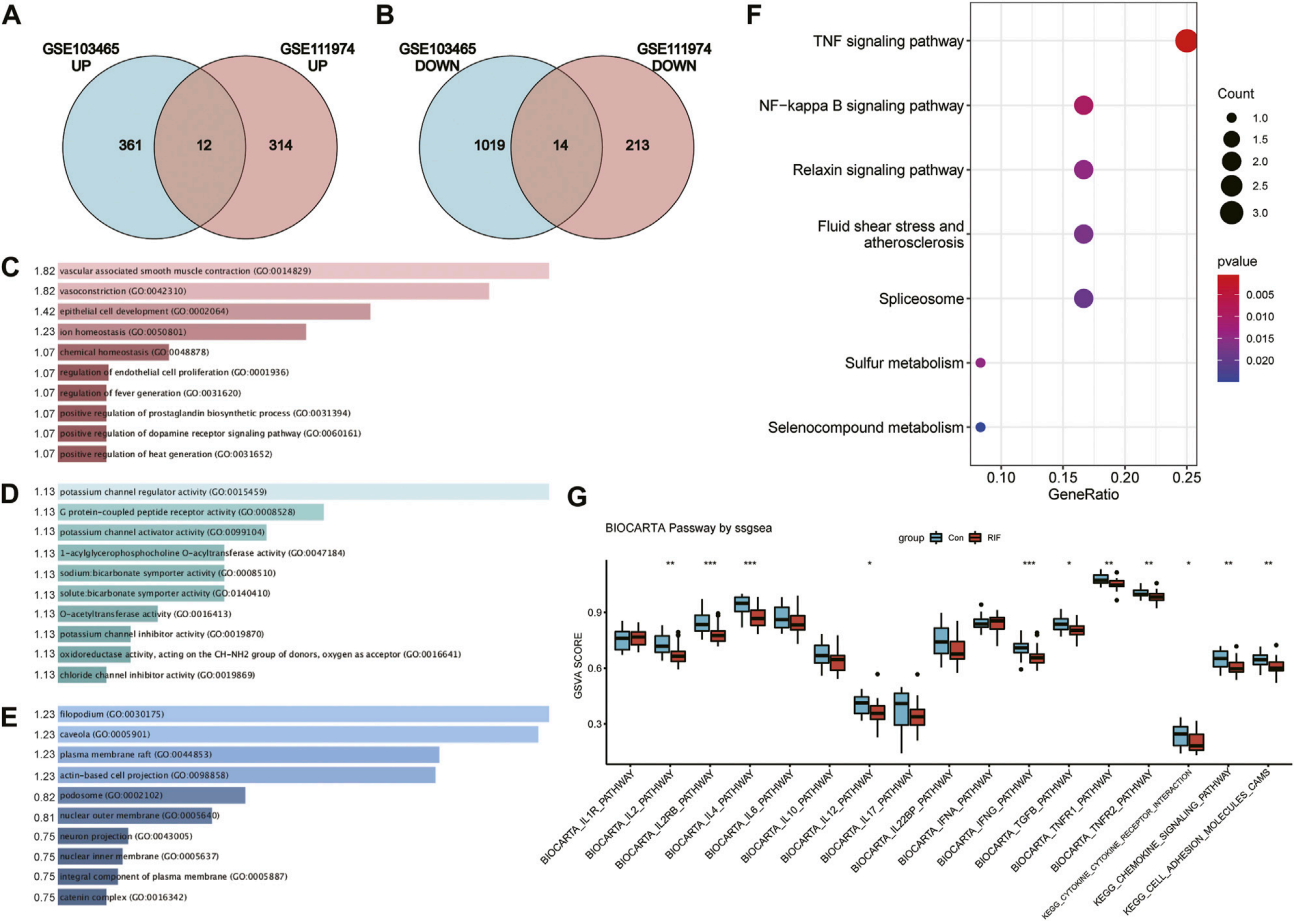
**(A, B)** Venn diagram of upregulated DEGs **(A)** and downregulated DEGs **(B)** from two datasets. **(C–E)** Bar graphs reflecting GO terms identification including Biological process **(C)**, molecular function **(D)** and cellular component **(E)** of common DEGs. The width represent the values of [−log (Adjusted *p*-value)], which are listed beside the bars. **(F)** Bubble chart visualizing KEGG pathway analysis of common DEGs. **(G)** Box plot of BioCarta and KEGG pathway analysis *via* ssGESA.

Subsequently, venn diagrams were presented in Figure 3 to illustrate the overlap of DEGs from the two datasets. As presented in Figure 3, we finally identified 26 common DEGs containing 12 upregulated and 14 downregulated genes. The details of the common DEGs were displayed in Table 2. We visualized the GO terms and KEGG/BioCarta pathways of common DEGs in Figure 3, for the further understanding of biological functions. The biological processes analysis suggested that common DEGs mainly participated in vascular associated smooth muscle contraction and vasoconstriction (Figure 3C). Molecular function subsection indicated that common DEGs were associated with potassium channel regulator activity (Figure 3D). Predominate cellular components consisting of products by common DEGs were filopodium and caveola (Figure 3E). According to KEGG pathway database in Figure 3F, common DEGs mainly took a part in TNF and nuclear factor kappa B (NF-κB) signaling pathway. As pointed out by BioCarta pathway enrichment in Figure 3G, the inflammation associated pathways including interleukin (IL)-4, inerferon (IFN)-γ, IL-2 receptor β chain (IL2RB), IL-2, tumor growth factor (TGF) -β, tumor necrosis factor receptor (TNFR)-1, TNFR-2, and IL-12 pathways were dramatically downregulated in RIF group. Although the levels of IL-6 and IL-10 pathways didn’t have a significant difference in two groups, we could still find a decreasing trend in RIF group. The specific data of pathway enrichment was displayed in Supplementary Table S1.

**TABLE 2:** Log FC and *p*-value of common DEGs.

Upregulated genes				
Gene.Symbol	Log FC		*p*.Value	
	GSE103465	GSE111974	GSE103465	GSE111974
C20orf96	3.01	1.35	6.50E-04	4.16E-07
EHF	2.53	1.76	9.91E-03	1.44E-10
HOXA6	2.42	1.20	4.52E-02	5.96E-08
KLHL31	1.07	1.34	4.63E-02	1.84E-04
LGR5	1.62	1.14	2.22E-02	3.51E-05
LRRC26	1.02	1.58	3.50E-02	1.51E-02
MTL5	1.43	1.08	4.85E-02	2.50E-03
PTGS2	1.23	1.40	2.01E-02	3.57E-04
SLC4A7	2.11	1.14	4.85E-02	3.35E-03
SNORD89	3.89	1.04	8.99E-04	2.74E-06
SP9	1.31	1.00	2.96E-02	2.02E-14
WNK4	1.46	2.15	1.21E-02	5.76E-06

Downregulated genes				
Gene.Symbol	Log FC		*p*.Value	
	GSE103465	GSE111974	GSE103465	GSE111974
ACTA2	−2.62	−1.35	1.19E-03	2.98E-05
C20orf3	−1.67	−1.05	4.96E-02	3.51E-09
CAV2	−1.95	−1.20	1.92E-02	4.21E-08
CDH13	−3.01	−1.13	3.14E-04	5.57E-04
EDNRB	−1.63	−1.17	3.76E-02	4.46E-03
EFEMP1	−2.17	−1.12	4.91E-03	5.40E-03
FUS	−1.23	−1.38	1.48E-02	2.69E-10
GAS1	−2.08	−1.46	1.07E-03	3.87E-05
LIF	−1.36	−1.35	2.64E-02	1.82E-02
LPCAT1	−1.99	−1.25	5.85E-03	1.07E-10
MLPH	−2.07	−1.22	3.68E-02	2.72E-03
PAPSS2	−1.57	−1.62	1.58E-02	5.62E-06
SRSF7	−1.51	−1.01	9.72E-03	1.06E-08
VCAM1	−1.77	−1.37	2.24E-02	2.11E-06

### PPI network and hub genes analysis

**FIGURE 4:**
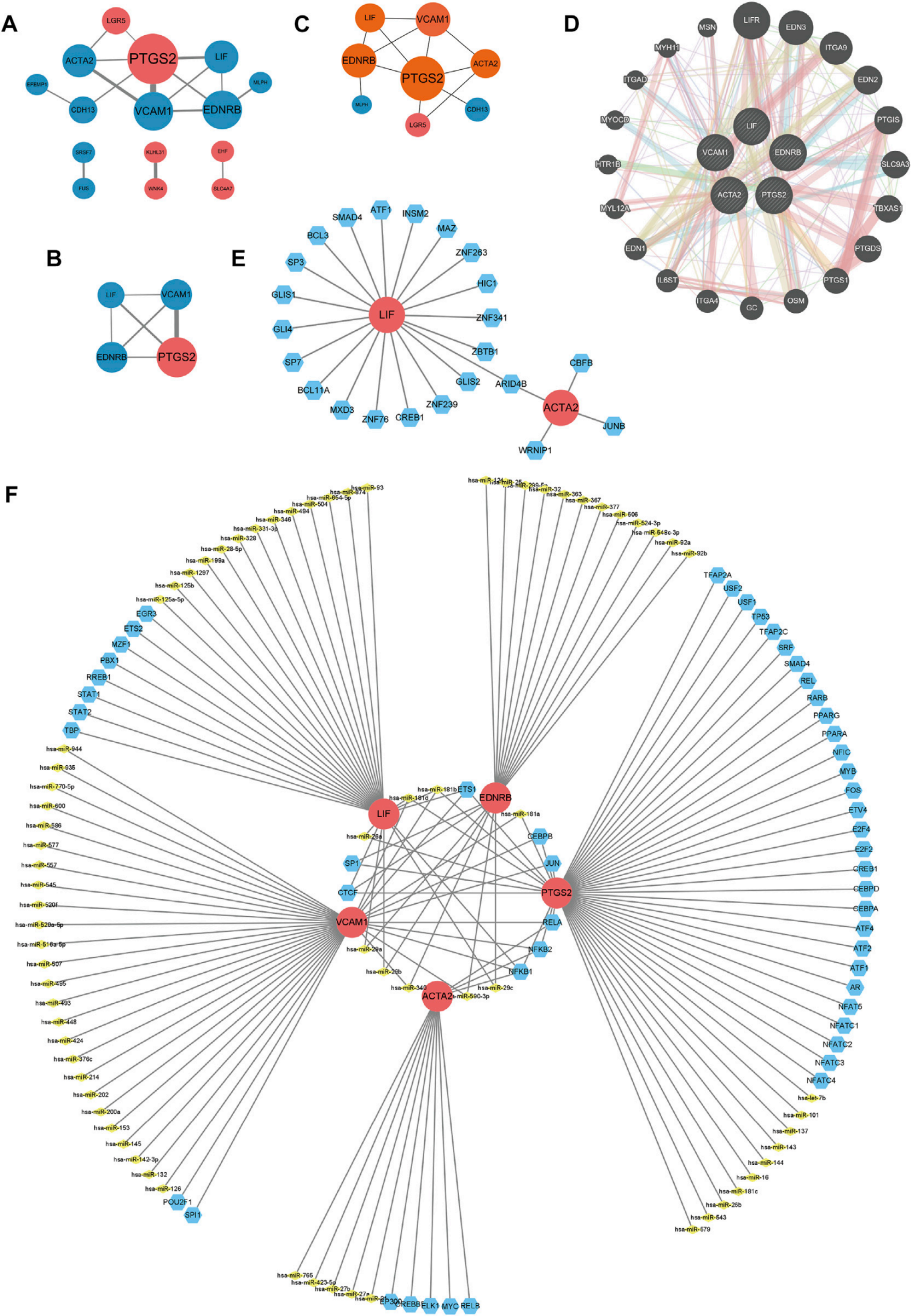
**(A–D)** PPI networks. **(A)** PPI network of 26 common DEGs. **(B)** Key modules of common DEGs. Larger nodes means higher degree scores. Thicker lines represent higher combined scores. Red nodes refer to upregulated genes, while blue ones are downregulated genes. **(C)** Display of hub genes, which are in orange nodes. **(D)** Interactions of hub genes and related genes. Pink lines represent physical interactions, purple lines refer to co-expression, orange lines direct to predicted interactions, blue lines mean co-localization, and green lines point to genetic interaction, cyan lines illustrate pathways, and yellow lines are shared protein domains. **(E)** Network for TF-gene interaction with hub genes. The highlighted red color node represents the hub genes and other blue nodes represent TF-genes. The TF-miRNA coregulatory network. The nodes red color are the hub genes, a yellow node represents miRNA and other blue nodes indicate TF-genes.

The PPI networks of common DEGs built by STRING database and Cytoscape software, which contained 15 nodes and 32 edges, as picturized in Figure 4A. In Figure 4B, we displayed the key PPI network *via* network gene clustering analysis. It was exhibited in Figure 4C that hub genes we identified were *Prostaglandin-endoperoxide synthase (PTGS) 2*, *Vascular cell adhesion molecule 1 (VCAM1)*, *Endothelin receptor type B (EDNRB)*, *Actin alpha 2 (ACTA2)*, and *Leukaemia inhibitory factor (LIF)*. The networks of hub genes and their relative genes from GeneMANIA (Figure 4D) indicated that those hub genes had a strong relationship with *Leukemia inhibitory factor receptor (LIFR), endothelin (EDN) 3, integrin subunit alpha (ITGA) 9, EDN2, prostacyclin synthase (PTGIS), solute carrier family nine isoform 3 (SLC9A3), Thromboxane synthase (TBXAS1), prostaglandin D2 synthase (PTGDS), PTGS1, oncostatin M (OSM), GC, ITGA4, interleukin six cytokine family signal transducer (IL6ST), EDN1, myosin light chain 12A (MYL12A), Serotonin receptor 1B (HTR1B), myocardin (MYOCD), integrin alpha D (ITGAD), Myosin heavy chain 11 (MYH11)* and *mesoporous silica nanoparticle (MSN)*. The specific interaction patterns could be obtained from Supplementary Table S2.

### TF-gene interaction and TF-miRNA coregulatory network

TF-gene interaction and TF-miRNA coregulatory network of common DEGs were identified with the aid of NetworkAnalyst, and were processed *via* Cytoscape. As shown in Figure 4E, the TF-gene interaction network consisted of 25 nodes and 24 edges. LIF was regulated by 19 TF-genes, while ACTA2 was regulated by four TF-genes, and the two hub genes shared a TF-gene called *AT-rich interaction domain (ARID4B)* (Figure 4E; Supplementary Table S3). TF-miRNA coregulatory network, as displayed in Figure 4F, contained 131 nodes and 151 edges, with 74 miRNAs and 52 TF-genes being collected. Among the most interacted TF-genes in TF-miRNA coregulatory network, *NF-κB1* had the highest degree value of 4, *CCCTC-binding factor (CTCF)*, *E26 transformation specific-1 (ETS1)*, *RelA* and *NF-κB2* had eminent degree values of 3, *specificity protein 1 (SP1)*, *Jun* and *CCAAT/enhancer-binding protein beta (CEBPB)* have relatively higher degree values of 2 (Figure 4F; Supplementary Table S4). Among the most interacted TF-miRNAs in TF-miRNA coregulatory network, hsa-miR-181b and hsa-miR-181d had relatively higher degrees values of 3, and the degree values of hsa-miR-181a, hsa-miR-340, hsa-miR-590-3p, hsa-miR-26a, hsa-miR-29a, hsa-miR-29b and hsa-miR-29c were 2 (Figure 4F; Supplementary Table S4). Considering the role of aforementioned TF-genes and TF-miRNAs, especially *NF-κB*, *Jun*, *CEBPB*, hsa-miR-181 and miR-miR-29 in inflammation, we believed that the inflammation could affect RIF also in a transcription level.

### Immune infiltration analysis

**FIGURE 5:**
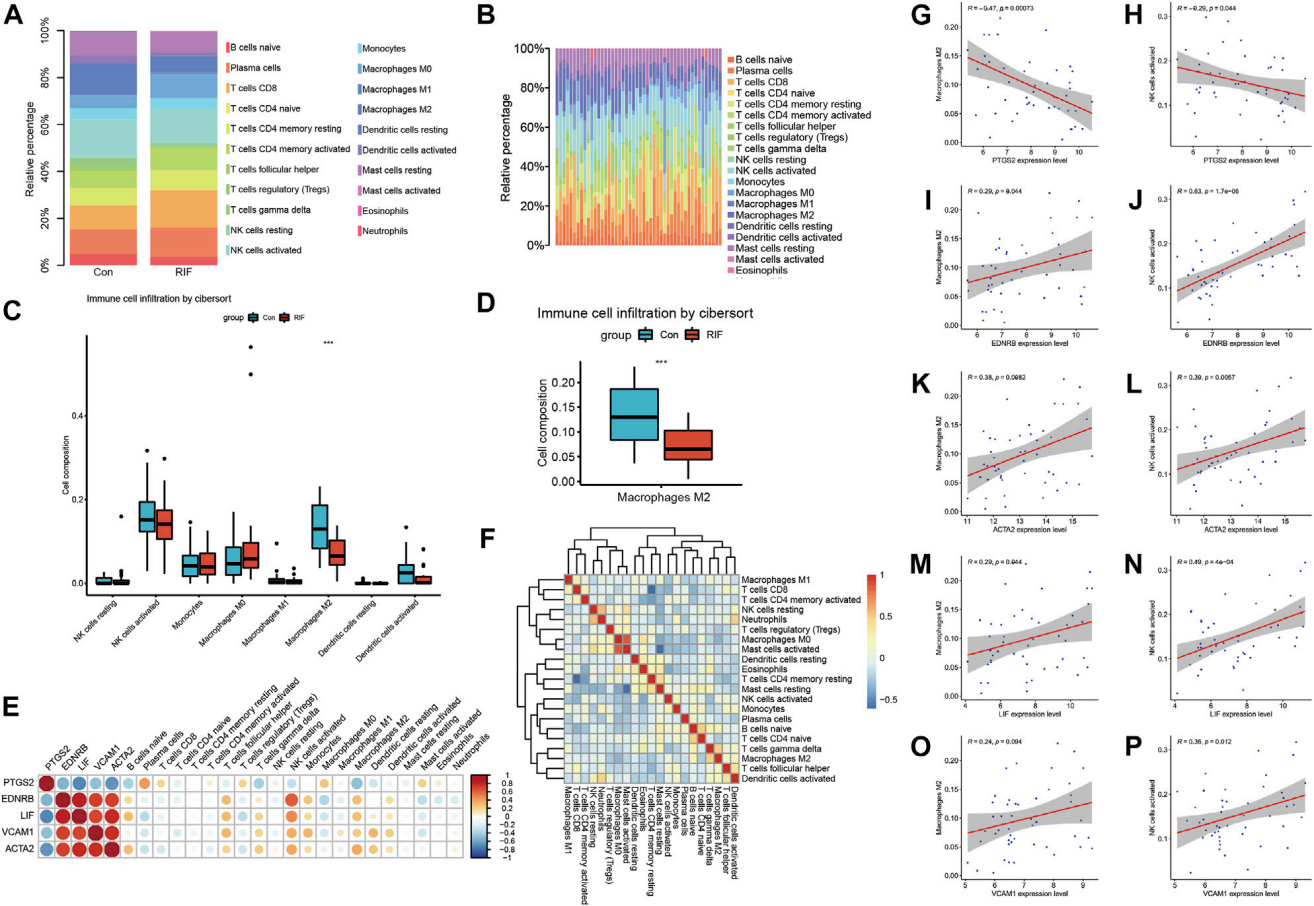
The immune infiltration analysis. **(A, B)** Immune infiltration analysis for 21 types of immune cell composition of endometrial tissue from RIF versues control group. **(C)** Box plot reveals the composition of eight types of immune cells in RIF and control group. **(D)** Box plot refers to the amount of M2 macrophages in RIF and control group. **(E)** Heat map exhibit the association of hub genes between hub genes and hub genes between immune cells. **(F)** Heat map reveals the relation of immune cells enriched. **(G,I,K,M,O)** Correlation scatter plots between expression of PTGS2/EDNRB/ACTA2/LIF/VCAM1 and Macrophages M2. **(H,J,L,N,P)** Correlation scatter plots between expression of PTGS2/EDNRB/ACTA2/LIF/VCAM1 and NK cells activated.

As presented in Figure 5, the percentage of macrophage M2, γδT cell and dendritic cells activated in RIF group were less than that in control group. The percentage of macrophage M2 in two groups was individually shown in Figure 5D. Although the levels of NK cells activated didn’t have significant differences in two groups, we could still find a decreasing trend of NK cells activated in RIF group, and larger scale of samples are required (Figure 5C). The result might be significant if there was a larger sample size. Subsequently, we executed the relationships between hub genes and hub genes, hub genes and infiltrated immune cells, infiltrated immune cells and immune cells (Figure 5). The results showed that hub genes have a tight relationship with the amount of macrophage M2 and NK cell activated. Therefore, we then excavated the association of hub genes expression and above mentioned two immune cells. As exhibited in Figure 5, the expression of PTGS2 had a negative correlation with macrophage M2 and NK cell activated (*p* < 0.05), while EDNRB, ACTA2 and LIF were positively correlated with macrophage M2 and NK cell activated (*p* < 0.05). VCAM1 had a positive relationship with NK cells activated (*p* < 0.05), although it did not have a significant relationship with macrophage M2 (*p* = 0.094), we could still observe a positive trend.

### Identification of RIF subgroups

**FIGURE 6:**
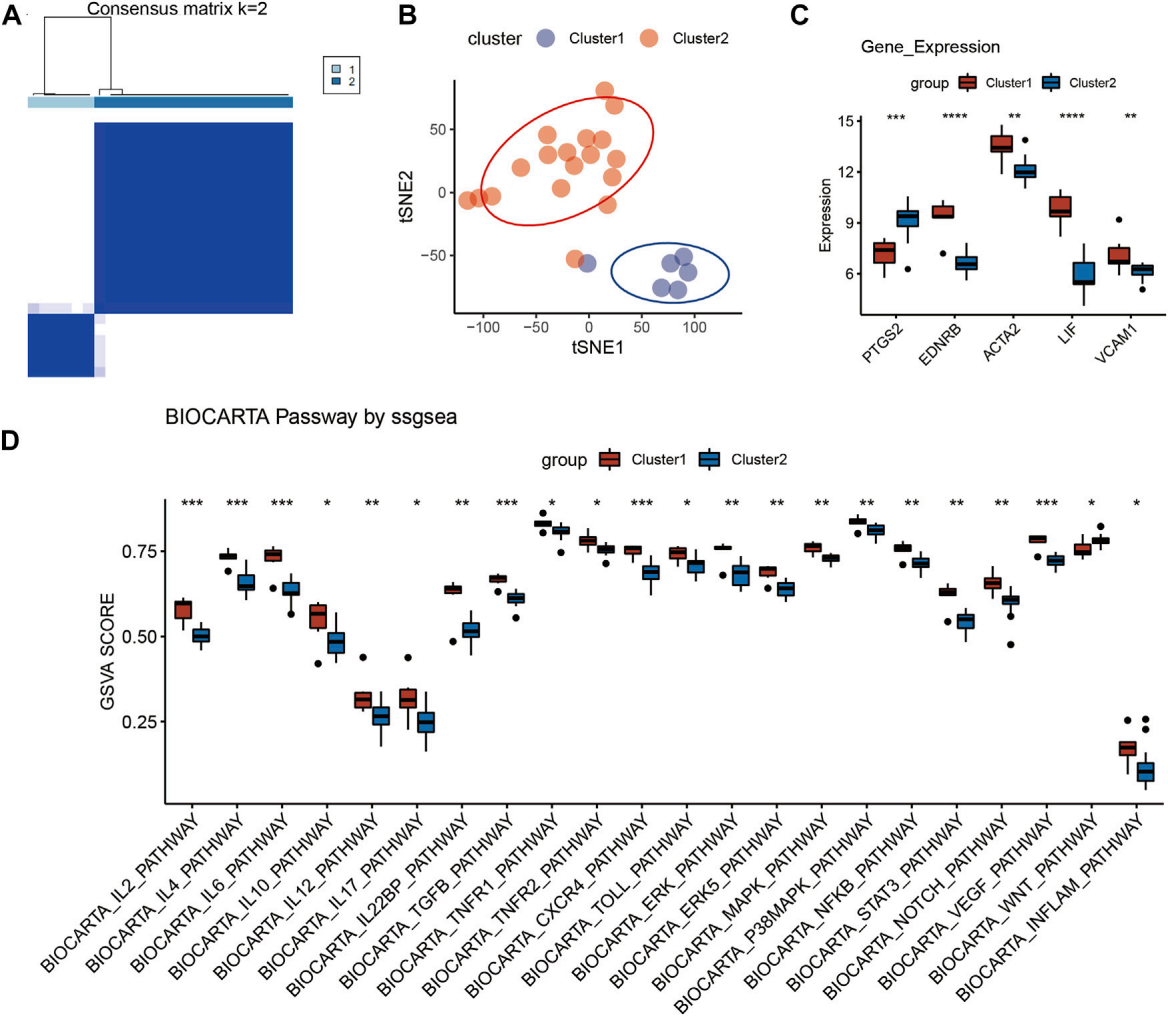
Identification of RIF subgroup in GSE111974. **(A)** Reordered consensus matrix on RIF compendium, by applying k-medoids with k = 2. **(B)** t-distributed Stochastic Neighbor Embedding (t-SNE) reduces the dimensions of a multivariate dataset. **(C)** Box plot reveals the expression of five hub genes in the two clusters. **(D)** Box plot of BioCarta pathway analysis in the two clusters *via* ssGESA.

The RIF individuals in GSE111974 could be grouped into 2 clusters *via* hub genes, which was displayed in Figure 6A, with a relatively larger cluster two and smaller cluster 1. Then the sample clustering condition in Figure 6B showed that the 2 clusters could be divided to two separate sections in the quadrant, which meant that there were remarkable differences between the two clusters. As shown in Figure 6C, the expression of PTGS2 was significantly higher in cluster 2, while the expression levels of *EDNRB*, *ACTA2*, *LIF*, and *VCAM1* were markedly decreased in cluster 2. The pathways which exhibited anti-inflammatory effect, such as IL-2, IL-4, IL-6, IL-10, TGF-β, TNFR2, and extracellular signal-regulated kinase 5 (ERK5) pathways were dramatically downregulated in cluster 2 (Figure 6D). Besides, Wnt pathway, mainly exerted pro-inflammatory effect, was significantly activated in cluster two compared with cluster 1 (Figure 6D). And other pathways like IL-12, IL-22 binding protein (IL-22 BP), Toll, extracellular signal-regulated kinase (ERK), IL-17, TNFR1, chemokine C-X-C motif ligand 4 (CXCR4), mitogen-activated protein kinases (MAPK), p38 MAPK, NF-κB, signal transducer and activator of transcription 3 (STAT3), Notch, vascular endothelial growth factor (VEGF) and Inflam pathways also had remarkable downregulations in cluster 2 (Figure 6D). Therefore, it was clear that the five hub genes could separate RIF patients into 2 clusters, with one presented a typical inflammatory environment in uterus (cluster 2) and another had atypical inflammatory responses (cluster 1) (Figure 6). Inflammatory alteration may contribute to RIF, but this cant explain all the patients, especially individuals in cluster 1, whose pathogenesis has been rarely discussed up to now. In future work, RIF individuals can be grouped by the five hub genes, and we can investigate specific etiology and accurate treatment methods for each group.

### ROC curve analysis

**FIGURE 7:**
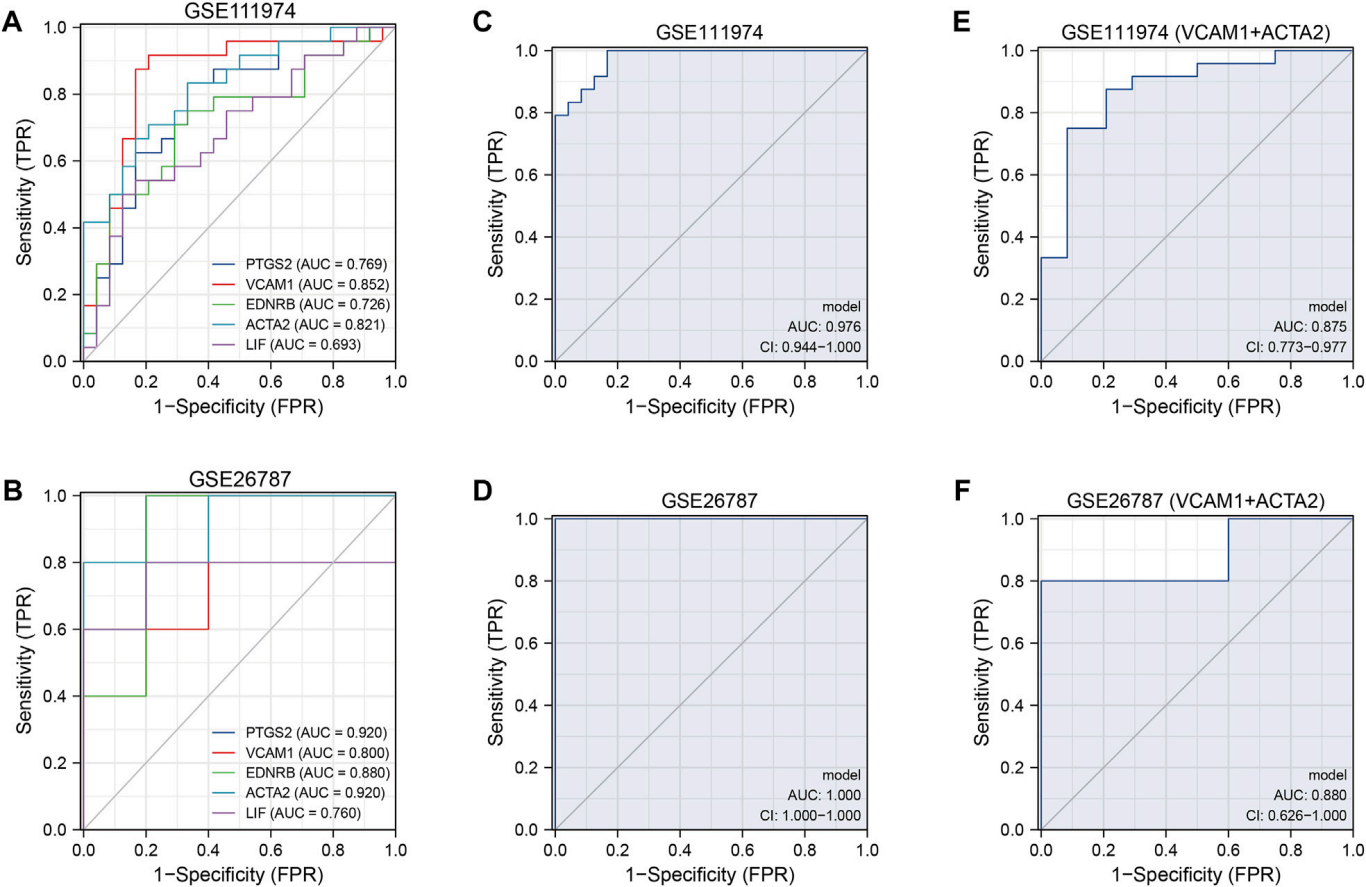
The ROC curves of the diagnostic model. **(A, B)** The diagnostic ROC curves of independent five hub genes in GSE111974 **(A)** and GSE26787 **(B)**. **(C, D)** The diagnostic ROC curves of combined five hub genes in GSE111974 **(C)** and GSE26787 **(D)**. **(E, F)** The diagnostic ROC curves of combined VCAM1 and ACTA2 in GSE111974 **(E)** and GSE26787 **(F)**.

In GSE111974, we found that the five hub genes showed important values in the diagnosis of RIF independently, especially VCAM1 and ACTA2, which had an AUC of 0.852 and 0.821, respectively (Figure 7A). The results were verified in GSE26787, with a high AUC (AUC>0.7) in each hub genes (Figure 7B). Combined the five hub genes, the AUC reached 0.976 when combined the five hub genes in GSE111974 (Figure 7C), while in GSE26787, the AUC was 1.0 (Figure 7D). Combined VCAM1 with ACTA2, the AUC achieved 0.875 in GSE11974 (Figure 7E) and 0.88 in GSE26787 (Figure 7F). To sum up, the hub genes we identified the five hub genes had good diagnostic values in RIF.

### Identification of potential drugs

From DSigDB database, we selected 10 drugs who had the minimal adjust *p*-value, they were: Simvastatin CTD 00007319, nimesulide CTD 00000666, probucol CTD 00006616, hesperidin CTD 00006087, Nebivolol CTD 00002249, progesterone CTD 00006624, Sphingosine 1-phosphate CTD 00002508, Hydroxytyrosol CTD 00000267, Ici 118,551 CTD 00001255, bisindolylmaleimide IX CTD 00002617 (Table 3). Among the 10 candidate drugs, progesterone CTD 00006624 could interact with all the five hub genes, and simvastatin CTD 00007319 had an interaction with the five hub genes except EDNRB.

**TABLE 3:** Suggested top drug compounds for RIF.

Term	*p*.Value	Adjusted p.Value	Genes
simvastatin CTD 00007319	2.59E-07	1.64E-04	ACTA2; VCAM1; LIF; PTGS2
nimesulide CTD 00000666	4.64E-07	1.64E-04	VCAM1; EDNRB; PTGS2
probucol CTD 00006616	3.30E-06	7.78E-04	ACTA2; VCAM1
hesperidin CTD 00006087	7.64E-06	9.45E-04	VCAM1; PTGS2
Nebivolol CTD 00002249	7.64E-06	9.45E-04	VCAM1; PTGS2
progesterone CTD 00006624	8.01E-06	9.45E-04	ACTA2; VCAM1; EDNRB; LIF; PTGS2
Sphingosine 1-phosphate CTD 00002508	1.50E-05	1.19E-03	VCAM1; PTGS2
Hydroxytyrosol CTD 00000267	1.50E-05	1.19E-03	VCAM1; PTGS2
Ici 118,551 CTD 00001255	1.75E-05	1.19E-03	ACTA2; PTGS2
bisindolylmaleimide IX CTD 00002617	1.75E-05	1.19E-03	ACTA2; PTGS2

## Discussion

In the current study, we tried to find biological changes contributing to the pathogenesis of RIF *via* gene profiling. Benefited from the combination of two microarray datasets GSE103465 and GSE111974, our results are more effective and reliable. We found that the disturbed inflammation regulation plays a key role in the pathogenesis of RIF, and abnormal uterine muscle contraction and vascularity also contribute to RIF. Although the five hub genes we identified have been discussed in previous studies, the molecular mechanism isn’t fully understood. We first revealed the interaction existed among the five hub genes, which associated with the disturbed inflammation regulation, uterine muscle contraction and vascularity in RIF. Of importance, the subgroup identification revealed that a small number of patients have atypical phenotypes (cluster 1), which may be the reason for the poor prognosis of RIF. It hints us that RIF individuals can be grouped by the five hub genes, and we can investigate specific etiology and accurate treatment methods for each group in future work. Additionally, the drug prediction revealed the potential drug molecules, which sheds new light on the treatment of RIF.

Inflammation is essential in various pathophysiological processes and diseases including cancer, allergic diseases, congenital diseases and so on (Gao et al., 2021). The role of inflammation in pregnancy has been argued for decades, of which importance is beyond doubt (Mor et al., 2011). Although a specific local pro-inflammatory environment is necessary for embryo implantation, excessive inflammatory response can also be harmful (Mor et al., 2011; Mekinian et al., 2016). Anti-inflammatory cytokines and cells perform multiple functions during normal pregnancies, such as promoting placental formation and angiogenesis, and modulating trophoblast differentiation and invasion (Chatterjee et al., 2014). Combining with results of current study, we believe that the ruined balance of pro-inflammatory and anti-inflammatory factors can ultimately lead to implantation failure.

KEGG pathway enrichment of common DEGs indicates that TNF and NF-κB signaling pathways play a major part in RIF. Canonical NF-κB pathway activation responds to a diversity of external stimuli involved in inflammatory and immune response, *via* inducing the expression of pro-inflammatory cytokines including TNF-α (Yu et al., 2020). Disturbed expression of NF-κB has been reported in women suffering from infertility, and gene polymorphism of NF-κB is noted to be related to RIF (Luo et al., 2016). Detectable TNF-α and NF-κB from feto-maternal surface are vital characters of successful implantation (Mor et al., 2011; Ersahin et al., 2016), but the overexpression of TNF-α and NF-κB also has adverse effect on implantation and lead to RIF (Ersahin et al., 2016; Mekinian et al., 2016). Moreover, Duan and colleagues found that TNF was the cytokine having the strongest correlation with all hub genes in their research on RIF (Duan et al., 2022). The afore-mentioned evidences suggest that NF-κB and TNF signaling pathways are essential in implantation, and should be cautiously controlled in an appropriate range. KEGG pathway calculated by ssGESA suggests that cytokine-cytokine receptor interaction pathway has a significant downregulation in RIF group. KEGG pathway calculated by ssGESA suggests that IL-2, IL-2 receptor beta (IL-2RB), IL-4, IL-12, IFN-γ, TGF-β, TNFR1, TNFR2, cytokine-cytokine receptor interaction, chemokine signaling pathway and cell adhesion molecules cams had significant downregulation in RIF group. Among the aforementioned pathways, IL-2RB, IL-4 and IFN-γ pathways showed the most remarkable decline (*p* < 0.001). IL-2 RB can induces growth potential for endometrial glandular epithelial cells, and its hypermethylation and downregulation has been found in ovarian endometriosis (Kusakabe et al., 2009; Zhang et al., 2022). IL-4 is always considered as a cytokine involved in anti-inflammatory effect, and is also a vital mediator of fetal tolerance in successful implantation and pregnancy (Feghali and Wright, 1997; Liang et al., 2015; Mekinian et al., 2016). IFN-γ, as a pro-inflammatory cytokine, has been reported to participate in uterine vascular modification and successful implantation (Feghali and Wright, 1997). The high ratios of IFN-γ/IL-4, IFN-γ/IL-10 and IFN-γ/TGF-β have been observed in the RIF and associated with adverse outcome of implantation (Liang et al., 2015). As pointed out by BioCarta pathway enrichment, the cytokine associated pathways, such as IL-2, IL-4, IL-6, CXCR4, and VEGF pathways, are dramatically downregulated (*p* < 0.001) in cluster 2, which contains the majority of RIF patients. Therefore, appropriate inflammatory activation is the crux of successful implantation.

Cytokines participating in implantation can be secreted by the endometrial cells and immune cells recruited to the position of implantation, 65%–70% of these cells are uterine-specific NK cells, and 10%–20% are macrophages (Mor et al., 2011). Depletion of these immune cells has deleterious effects on implantation, deciduation and placental development (Mor et al., 2011). In current immune infiltration analysis of cell composition, M2 macrophages and NK cells are memorably lower in RIF group than that in control group. Uterine NK cells have long been acknowledged to be essential in deciduation for its role in endometrial vascularity (Mor et al., 2011). M2 macrophages can be polarized by anti-inflammatory cytokines such as IL-4, IL-10, and TGF-β, conversely, they can produce large quantities of IL-10 and TGF-β to suppress the inflammation, for the purpose of tissue repair, remodeling and vasculogenesis (Shapouri-Moghaddam et al., 2018). The depletion of M2 macrophages in mice has been demonstrated to be a cause of implantation failure (Ono et al., 2020).

Consistent with previous studies, we find that the imbalance of pro-inflammatory and protective factors leads to a disordered immune environment in uterus, results in abnormal vascularity and muscle contraction, and finally bring about RIF. But our analysis revealed a more comprehensive inflammatory pathway spectrum in RIF. The function of identified hub genes (*PTGS2*, *VCAM1*, *EDNRB*, *ACTA2*, and *LIF*) also highlights the importance of inflammation, uterine muscle contraction and vascularity in RIF.

PTGS2 is synthesized at very low levels under normal conditions, but can be stimulated by specific events and is responsible for the prostanoid biosynthesis under inflammation (Vane et al., 1998). Most of the stimuli that induce PTGS2 are those associated with inflammation, such as TNF-α, while IL-4 presented an inhibitory impact on PTGS2 (Vane et al., 1998). For decades, PTGS2 has been widely considered to be an indispensable molecule in female reproductive process including ovulation, fertilization, implantation and embryo development (Anamthathmakula and Winuthayanon, 2021). However, our analysis showed that there was a significant increase of PTGS2 in RIF, suggesting that the over expression of PTGS2 may also be unfavorable for implantation. Rodent and simian models have revealed that premature uterine contraction may be associated with the activation of prostaglandin signaling, since PTGS2 inhibitors can dampen cytokine induced uterine contractility (Sadowsky et al., 2000; Mackler et al., 2003; Cirillo et al., 2007; Orsi and Tribe, 2008). Additionally, high-frequency uterine contraction at the time of embryo transfer had an adverse impact on implantation rates in IVF-ET (Fanchin et al., 1998). Our analysis provided a new sight that the overexpression of PTGS2 may also impair the endometrial receptivity during WOI, *via* leading to activated inflammatory cascade and uterine muscle contraction. As a consequence, the level of PTGS2 in endometrium needs a more precise regulation.

*VCAM1* is a downregulated hub gene in our analysis. *VCAM1* is a NF-κB target gene induced by TNF-TNFR1 signaling pathway and IL-4 (Kong et al., 2018). As an adhesion molecule, VCAM1 is associated with epithelial cells activation, neutrophil recruitment and aggravated creatine kinase (He et al., 2021; Mao et al., 2021). In reproductive system, VCAM1 is known to appear in endometrial side of decidual stromal cells (Bai et al., 2014). Significantly lower expression of VCAM1 in endometrium at the peri-implantation stage is associated with unexplained infertility and implantation failure after IVF(Konac et al., 2009). Uterine VCAM1 expression is essential for conceptus-uterine endometrium adhesion and early placental development, so mutations or deficiencies of the *VCAM1* may contribute to a series of human placental insufficiencies. (Gurtner et al., 1995; Bai et al., 2014). In Shang and colleague’s research, *VCAM1* was also regarded as a key gene in miRNA-mRNA interaction network of RIF (Shang et al., 2022). Result of our analysis is tied well with the previous studies, so we believe that VCAM1 is a vital molecule in implantation and has the potential to be a biomarker in RIF.

EDNRB widely locates in vascular endothelium of many human tissues including placenta (Gram et al., 2017). The expression of EDNRB was increased in the perivascular and vascular cells of branching vessels during the late secretory phase (Keator et al., 2011). And EDNRB was constantly expressed during pregnancy including peri-implantation phase (Gram et al., 2017). What intrigues us is that during prepartum luteolysis, elevated expression of the EDN receptors in placenta strongly resembles the placental localization of PGs family members (e.g., PTGS2) in dogs (Kowalewski et al., 2010; Gram et al., 2014). In addition, the elevation of EDN1 during normal prepartum luteolysis and antigestagen-induced parturition/abortion is associated with increased PGs output in dogs (Kowalewski et al., 2010; Gram et al., 2014). In brief, EDNRB is strongly associated with all stages of pregnancy including implantation and involved in the signaling cascade of leukocyte recruitment and PGs synthesis, loss of EDNRB has the probability to lead to implantation failure.

Actin alpha 2 (ACTA2) is a smooth muscle actin predominately participated in vascular contractility and blood pressure homeostasis (Maglott et al., 2011). Expressed in uterine myocytes, ACTA2 is associated with uterine muscle contraction and uterine remodeling in pregnancy (Cooper and Brown, 2017). Women with ACTA2 mutations may be more likely to suffer from uterine muscle dysfunction and hemorrhage, according to a case report by Kylie and colleagues (Cooper and Brown, 2017). Our study revealed the potential and indispensable role of ACTA2 in implantation *via* impacting the function of uterine muscle.

As a member of IL-6 cytokine family, LIF has been reported to exhibit both pro-inflammatory and anti-inflammatory effects (Gadient and Patterson, 1999). Like PTGS2, LIF is also strongly elevated by TNF and downregulated by IL-4 (Gadient and Patterson, 1999). Conversely, LIF induces the production of pro-inflammatory cytokines (Gadient and Patterson, 1999). LIF is viewed as a potential predictor of fertility after IVF, considering that high expression levels of LIF in endometrium during the mid-luteal phase are relevant to a higher rate of pregnancy success in women underwent IVF (Serafini et al., 2008). The mechanisms of LIF in modulating implantation have been well discussed. LIF influences endometrial receptivity through inducing decidualization, elevating IL-6 and IL-15 levels in decidual cells, and recruiting leukocytes during WOI (Kimber, 2005; Shuya et al., 2011). LIF also has the ability to accelerate the transformation of endometrial macrophages into an anti-inflammatory phenotype *via* LIF-signal transducer and activator of transcription (STAT) pathways (Brinsden et al., 2009; Dallagi et al., 2015).

TF-genes are reactors for the gene expression regulation, through binding with targeted genes and miRNAs (Zhang et al., 2015). Among the regulators, ARID4B has a significant interaction, which has been demonstrated to be a regulator of male fertility (Wu et al., 2013). Regulatory biomolecules work as potential biomarkers in plenty of complex diseases. As shown in TF-genes and TF-miRNA coregulatory network, NF-κB1, NF-κB2, RelA, CEBPB, miR-181, and miR-29 are important in RIF. NF-κB, RelA (a subunit of NF-κB) and CEBPB are both key regulators in inflammation (Zahid et al., 2020; Zhao et al., 2020). MiRNAs can regulate various target genes in numerous biological processes and diseases, such as endometrial cancer (Ni et al., 2022). It has been reported that miR-181 family present a central role in vascular inflammation (Sun et al., 2014). MiR-181b inhibits expression of VCAM1, and serves as an inhibitor of downstream NF-κB signaling pathway (Sun et al., 2012; Sun et al., 2014). MiR-181d has the responsibility to regulate the acute stress response in thymocytes *via* targeting LIF (Sun et al., 2014). Combing mRNA microarray GSE111974 with miRNA microarray GSE71332, Ahmadi and colleagues constructed a circRNA-miRNA-mRNA network in RIF, and found that miR-29c might be a crucial miRNA, which is consistent with our analysis (Ahmadi et al., 2022). In addition, overexpression of miR-29 has been reported to impair endometrial receptivity by inhibiting the differentiation of endometrial stromal cells and regulating decidualization (Zhou et al., 2021).

We then used cluster analysis, ROC analysis and DSigDB database, in order to the identify the clinical diagnostic and therapeutic value of the five hub genes in RIF. Considering the results of RIF subgroup identification, the patients with RIF can be divided into two subgroups. Most patients (cluster2) have similar characteristics with RIF we previously believed, but there are still some patients have atypical phenotypes (cluster 1), which may be one of the reasons for the poor therapeutic effect of RIF. Therefore, our future research should investigate specific etiology and accurate treatment methods for each group. The ROC curve in GSE111974 shows that the five genes do make sense in diagnosis of RIF, and the result was furtherly verified in RIF group with another GEO datasets GSE26787. According to DSigDB database, the current study highlights the progesterone CTD 00006624 and simvastatin CTD 00007319 as two drug molecules that most hub genes interacted with. Progesterone is a necessary hormone for pregnancy, and its supplementation can prevent recurrent miscarriage and reduce implantation failure in IVT cycles (Nardo and Sallam, 2006). Simvastatin, a lipid regulating agent, also shows impact on polycystic ovary syndrome (PCOS), endometriosis and uterine fibrosis (Banaszewska et al., 2011; Taylor et al., 2017; Ali et al., 2018). Simvastatin has been reported to reduce the expression of neopterin in endometriosis, which is a marker of inflammation and immune system activation (Taylor et al., 2017). Simvastatin can also inhibit C-reactive protein in PCOS and suppress fibroid proliferation and extracellular matrix production in uterine fibrosis (Banaszewska et al., 2011; Ali et al., 2018).

However, this study also has limitations. The sample size of our study is still insufficient and our research lacks of experimental validation. Non-etheless, we believe that the inflammatory regulation network as well as uterine muscle contraction and vascularity play an essential role in RIF.

## Conclusion

In present context, we accomplished DEGs analysis of endometrial tissue between RIF and control women in two datasets and identified 26 common DEGs. KEGG/BioCarta pathway and immune infiltration analysis determined inflammation associated pathways and cells might contribute to RIF. GO terms, five hub genes (*PTGS2*, *VCAM1*, *EDNRB*, *ACTA2*, and *LIF*) and related TF-gene and TF-miRNA interactions were identified, suggesting that inflammation, uterine muscle contraction and vascularity were key pathophysiological changes in RIF. Of interest, subgroup identification revealed that the patients with RIF can be divided into two subgroups, and a small number of patients have atypical phenotypes (cluster 1), which may be the reason for the poor prognosis of RIF. ROC curves and drugs affirmed the diagnostic and therapeutic values of hub genes. Those results may help us expand the understanding of RIF and may provide evidences for the treatment of RIF. Further researches should consider the underlying mechanisms of the inflammatory regulation as well as uterine muscle contraction and vascularity in RIF, and find meaningful diagnostic and treatment methods.

## Data Availability

The datasets presented in this study can be found in online repositories. The names of the repository/repositories and accession number(s) can be found in the article/Supplementary Material.
