# Supplementary material for: Gene profiling reveals the role of inflammation, abnormal uterine muscle contraction and vascularity in recurrent implantation failure
Source: Front Genet. 2023 Feb 24;14:1108805. doi: 10.3389/fgene.2023.1108805 (PMC9998698; doi:10.3389/fgene.2023.1108805)
Supplement: Supplementary file 1 [file Table1.DOCX]

**Supplementary Table 1** | Data of GO terms and KEGG pathway enrichment.

|  | Term | P-value | Adjusted P-value | Odds Ratio | Combined Score | Genes |
| --- | --- | --- | --- | --- | --- | --- |
| BP | vascular associated smooth muscle contraction (GO:0014829) | 4.53E-05 | 0.015 | 277.333 | 2774.054 | ACTA2;  EDNRB |
|  | vasoconstriction (GO:0042310) | 8.87E-05 | 0.015 | 184.861 | 1724.729 | ACTA2;  EDNRB |
|  | epithelial cell development (GO:0002064) | 3.36E-04 | 0.038 | 87.522 | 700.007 | ACTA2;  SLC4A7 |
|  | ion homeostasis (GO:0050801) | 6.91E-04 | 0.059 | 59.363 | 431.987 | SLC4A7;  WNK4 |
|  | chemical homeostasis (GO:0048878) | 3.21E-03 | 0.085 | 26.337 | 151.181 | SLC4A7;  WNK4 |
|  | regulation of endothelial cell proliferation (GO:0001936) | 6.33E-03 | 0.085 | 18.411 | 93.203 | CAV2;  CDH13 |
|  | regulation of fever generation (GO:0031620) | 6.48E-03 | 0.085 | 199.700 | 1006.182 | PTGS2 |
|  | positive regulation of prostaglandin biosynthetic process (GO:0031394) | 6.48E-03 | 0.085 | 199.700 | 1006.182 | PTGS2 |
|  | positive regulation of dopamine receptor signaling pathway (GO:0060161) | 6.48E-03 | 0.085 | 199.700 | 1006.182 | CAV2 |
|  | positive regulation of heat generation (GO:0031652) | 6.48E-03 | 0.085 | 199.700 | 1006.182 | PTGS2 |
| MF | potassium channel regulator activity (GO:0015459) | 1.92E-03 | 0.074 | 34.594 | 216.475 | LRRC26;  WNK4 |
|  | G protein-coupled peptide receptor activity (GO:0008528) | 5.06E-03 | 0.074 | 20.723 | 109.538 | EDNRB;  LGR5 |
|  | potassium channel activator activity (GO:0099104) | 6.48E-03 | 0.074 | 199.700 | 1006.182 | LRRC26 |
|  | 1-acylglycerophosphocholine O-acyltransferase activity (GO:0047184) | 7.78E-03 | 0.074 | 159.752 | 775.878 | LPCAT1 |
|  | sodium:bicarbonate symporter activity (GO:0008510) | 7.78E-03 | 0.074 | 159.752 | 775.878 | SLC4A7 |
|  | solute:bicarbonate symporter activity (GO:0140410) | 7.78E-03 | 0.074 | 159.752 | 775.878 | SLC4A7 |
|  | O-acetyltransferase activity (GO:0016413) | 1.04E-02 | 0.074 | 114.097 | 521.462 | LPCAT1 |
|  | potassium channel inhibitor activity (GO:0019870) | 1.16E-02 | 0.074 | 99.830 | 444.560 | WNK4 |
|  | oxidoreductase activity, acting on the CH-NH2 group of donors, oxygen as acceptor (GO:0016641) | 1.16E-02 | 0.074 | 99.830 | 444.560 | VCAM1 |
|  | chloride channel inhibitor activity (GO:0019869) | 1.29E-02 | 0.074 | 88.733 | 385.851 | WNK4 |
| CC | filopodium (GO:0030175) | 2.57E-03 | 0.058 | 29.640 | 176.783 | ACTA2;  VCAM1 |
|  | caveola (GO:0005901) | 2.75E-03 | 0.058 | 28.615 | 168.759 | CAV2;  CDH13 |
|  | plasma membrane raft (GO:0044853) | 5.06E-03 | 0.058 | 20.723 | 109.538 | CAV2;  CDH13 |
|  | actin-based cell projection (GO:0098858) | 5.18E-03 | 0.058 | 20.466 | 107.697 | ACTA2;  VCAM1 |
|  | podosome (GO:0002102) | 1.68E-02 | 0.151 | 66.540 | 272.012 | VCAM1 |
|  | nuclear outer membrane (GO:0005640) | 2.06E-02 | 0.155 | 53.224 | 206.625 | PTGS2 |
|  | neuron projection (GO:0043005) | 3.46E-02 | 0.178 | 4.581 | 15.412 | MLPH;  CDH13;  PTGS2 |
|  | nuclear inner membrane (GO:0005637) | 3.58E-02 | 0.178 | 29.551 | 98.406 | PTGS2 |
|  | integral component of plasma membrane (GO:0005887) | 3.68E-02 | 0.178 | 3.044 | 10.049 | LRRC26;  EDNRB;  VCAM1;  CAV2;  LGR5 |
|  | catenin complex (GO:0016342) | 3.96E-02 | 0.178 | 26.592 | 85.895 | CDH13 |
| KEGG | TNF signaling pathway | 4.05E-04 | 0.023 | 23.771 | 185.715 | VCAM1;  LIF;  PTGS2 |
|  | NF-kappa B signaling pathway | 8.02E-03 | 0.151 | 16.235 | 78.341 | VCAM1;  PTGS2 |
|  | Relaxin signaling pathway | 1.21E-02 | 0.151 | 13.023 | 57.464 | ACTA2;  EDNRB |
|  | Sulfur metabolism | 1.29E-02 | 0.151 | 88.733 | 385.851 | PAPSS2 |
|  | Fluid shear stress and atherosclerosis | 1.40E-02 | 0.151 | 12.066 | 51.530 | VCAM1;  CAV2 |
|  | Spliceosome | 1.61E-02 | 0.151 | 11.163 | 46.065 | FUS;  SRSF7 |
|  | Selenocompound metabolism | 2.19E-02 | 0.175 | 49.895 | 190.707 | PAPSS2 |
|  | African trypanosomiasis | 4.70E-02 | 0.273 | 22.153 | 67.721 | VCAM1 |
|  | Ether lipid metabolism | 6.18E-02 | 0.273 | 16.605 | 46.219 | LPCAT1 |
|  | Malaria | 6.30E-02 | 0.273 | 16.265 | 44.955 | VCAM1 |

**Supplementary Table 2** | Data of hub gene related genes analysis by GENEMANIA.

| Entity 1 | Entity 2 | Weight | Network group | Network |
| --- | --- | --- | --- | --- |
| PTGDS | VCAM1 | 0.0137 | Co-expression | Wang-Maris-2006 |
| OSM | PTGS2 | 0.0117 | Co-expression | Wang-Maris-2006 |
| OSM | EDN2 | 0.0122 | Co-expression | Wang-Maris-2006 |
| GC | LIF | 0.0172 | Co-expression | Wang-Maris-2006 |
| IL6ST | EDNRB | 0.0180 | Co-expression | Wang-Maris-2006 |
| MYH11 | ACTA2 | 0.0192 | Co-expression | Wang-Maris-2006 |
| MSN | MYL12A | 0.0060 | Co-expression | Wang-Maris-2006 |
| PTGS1 | ACTA2 | 0.0120 | Co-expression | Mallon-McKay-2013 |
| MYH11 | ACTA2 | 0.0139 | Co-expression | Mallon-McKay-2013 |
| MYH11 | PTGS1 | 0.0241 | Co-expression | Mallon-McKay-2013 |
| PTGS2 | LIF | 0.0190 | Co-expression | Roth-Zlotnik-2006 |
| LIFR | EDNRB | 0.0284 | Co-expression | Roth-Zlotnik-2006 |
| IL6ST | VCAM1 | 0.0135 | Co-expression | Roth-Zlotnik-2006 |
| MYOCD | ACTA2 | 0.0089 | Co-expression | Roth-Zlotnik-2006 |
| MYOCD | PTGIS | 0.0075 | Co-expression | Roth-Zlotnik-2006 |
| MYH11 | ACTA2 | 0.0137 | Co-expression | Roth-Zlotnik-2006 |
| MYH11 | MYOCD | 0.0110 | Co-expression | Roth-Zlotnik-2006 |
| OSM | EDN2 | 0.0050 | Co-expression | Ramaswamy-Golub-2001 |
| EDN1 | EDN2 | 0.0054 | Co-expression | Ramaswamy-Golub-2001 |
| MYH11 | ACTA2 | 0.0071 | Co-expression | Ramaswamy-Golub-2001 |
| PTGS2 | LIF | 0.0129 | Co-expression | Innocenti-Brown-2011 |
| LIFR | VCAM1 | 0.0146 | Co-expression | Innocenti-Brown-2011 |
| OSM | LIF | 0.0107 | Co-expression | Innocenti-Brown-2011 |
| OSM | PTGS2 | 0.0065 | Co-expression | Innocenti-Brown-2011 |
| IL6ST | LIFR | 0.0206 | Co-expression | Innocenti-Brown-2011 |
| MYH11 | PTGDS | 0.0111 | Co-expression | Innocenti-Brown-2011 |
| LIFR | VCAM1 | 0.0134 | Co-expression | Alizadeh-Staudt-2000 |
| PTGIS | EDN3 | 0.0054 | Co-expression | Dobbin-Giordano-2005 |
| ITGA4 | EDN3 | 0.0040 | Co-expression | Dobbin-Giordano-2005 |
| EDN2 | LIF | 0.0064 | Co-expression | Rieger-Chu-2004 |
| SLC9A3 | EDN2 | 0.0048 | Co-expression | Rieger-Chu-2004 |
| PTGS1 | EDN2 | 0.0079 | Co-expression | Rieger-Chu-2004 |
| HTR1B | EDN2 | 0.0103 | Co-expression | Rieger-Chu-2004 |
| EDN3 | VCAM1 | 0.0088 | Co-expression | Bild-Nevins-2006 B |
| PTGS1 | LIF | 0.0153 | Co-expression | Bild-Nevins-2006 B |
| GC | PTGIS | 0.0108 | Co-expression | Bild-Nevins-2006 B |
| HTR1B | SLC9A3 | 0.0076 | Co-expression | Bild-Nevins-2006 B |
| ITGAD | SLC9A3 | 0.0055 | Co-expression | Bild-Nevins-2006 B |
| PTGS2 | EDNRB | 0.0167 | Co-expression | Burington-Shaughnessy-2008 |
| ITGAD | VCAM1 | 0.0103 | Co-expression | Burington-Shaughnessy-2008 |
| MSN | MYL12A | 0.0138 | Co-expression | Burington-Shaughnessy-2008 |
| LIFR | PTGS2 | 0.0206 | Co-expression | Boldrick-Relman-2002 |
| ITGA9 | LIFR | 0.0218 | Co-expression | Boldrick-Relman-2002 |
| EDN1 | LIF | 0.0105 | Co-expression | Boldrick-Relman-2002 |
| EDN1 | PTGS1 | 0.0093 | Co-expression | Boldrick-Relman-2002 |
| PTGS2 | LIF | 0.0091 | Co-expression | Arijs-Rutgeerts-2009 |
| LIFR | VCAM1 | 0.0079 | Co-expression | Arijs-Rutgeerts-2009 |
| PTGDS | VCAM1 | 0.0033 | Co-expression | Arijs-Rutgeerts-2009 |
| PTGDS | LIFR | 0.0075 | Co-expression | Arijs-Rutgeerts-2009 |
| OSM | PTGS2 | 0.0114 | Co-expression | Arijs-Rutgeerts-2009 |
| ITGA4 | PTGDS | 0.0024 | Co-expression | Arijs-Rutgeerts-2009 |
| MYOCD | ACTA2 | 0.0195 | Co-expression | Arijs-Rutgeerts-2009 |
| MYH11 | ACTA2 | 0.0235 | Co-expression | Arijs-Rutgeerts-2009 |
| MYH11 | MYOCD | 0.0191 | Co-expression | Arijs-Rutgeerts-2009 |
| ITGAD | ITGA9 | 0.0143 | Co-expression | Jiang-de Kok-2017 |
| VCAM1 | ACTA2 | 0.0027 | Co-expression | Perou-Botstein-2000 |
| EDN3 | EDNRB | 0.0103 | Co-expression | Perou-Botstein-2000 |
| PTGIS | ACTA2 | 0.0039 | Co-expression | Perou-Botstein-2000 |
| PTGIS | PTGS2 | 0.0069 | Co-expression | Chen-Brown-2002 |
| PTGIS | EDN3 | 0.0100 | Co-expression | Chen-Brown-2002 |
| EDN1 | PTGIS | 0.0093 | Co-expression | Chen-Brown-2002 |
| MYH11 | ACTA2 | 0.0160 | Co-expression | Chen-Brown-2002 |
| MYH11 | PTGIS | 0.0093 | Co-expression | Chen-Brown-2002 |
| MSN | ACTA2 | 0.0119 | Co-expression | Chen-Brown-2002 |
| OSM | LIF | 0.0087 | Co-expression | Wang-Cheung-2015 |
| HTR1B | PTGDS | 0.0107 | Co-expression | Wang-Cheung-2015 |
| ITGA9 | LIF | 0.0127 | Co-expression | Wu-Garvey-2007 |
| OSM | SLC9A3 | 0.0049 | Co-expression | Wu-Garvey-2007 |
| IL6ST | LIFR | 0.0089 | Co-expression | Wu-Garvey-2007 |
| ITGAD | EDN2 | 0.0085 | Co-expression | Wu-Garvey-2007 |
| MYH11 | ACTA2 | 0.0342 | Co-expression | Wu-Garvey-2007 |
| MSN | ACTA2 | 0.0146 | Co-expression | Wu-Garvey-2007 |
| LIFR | PTGS2 | 0.0134 | Co-expression | Rosenwald-Staudt-2001 |
| LIFR | VCAM1 | 0.0103 | Co-expression | Rosenwald-Staudt-2001 |
| TBXAS1 | EDN3 | 0.0182 | Co-expression | Ross-Perou-2001 |
| PTGDS | EDN3 | 0.0137 | Co-expression | Ross-Perou-2001 |
| VCAM1 | ACTA2 | 0.0064 | Co-localization | Schadt-Shoemaker-2004 |
| PTGIS | ACTA2 | 0.0081 | Co-localization | Schadt-Shoemaker-2004 |
| PTGS1 | ACTA2 | 0.0068 | Co-localization | Schadt-Shoemaker-2004 |
| PTGS1 | PTGIS | 0.0119 | Co-localization | Schadt-Shoemaker-2004 |
| IL6ST | ACTA2 | 0.0092 | Co-localization | Schadt-Shoemaker-2004 |
| EDN1 | IL6ST | 0.0198 | Co-localization | Schadt-Shoemaker-2004 |
| HTR1B | GC | 0.0108 | Co-localization | Schadt-Shoemaker-2004 |
| LIFR | EDNRB | 0.0158 | Co-localization | Johnson-Shoemaker-2003 |
| TBXAS1 | VCAM1 | 0.0095 | Co-localization | Johnson-Shoemaker-2003 |
| PTGS1 | PTGIS | 0.0201 | Co-localization | Johnson-Shoemaker-2003 |
| IL6ST | EDNRB | 0.0176 | Co-localization | Johnson-Shoemaker-2003 |
| IL6ST | LIFR | 0.0266 | Co-localization | Johnson-Shoemaker-2003 |
| MYH11 | ACTA2 | 0.0070 | Co-localization | Johnson-Shoemaker-2003 |
| MYH11 | PTGS1 | 0.0075 | Co-localization | Johnson-Shoemaker-2003 |
| HTR1B | PTGS2 | 0.8001 | Genetic Interactions | IREF-SMALL-SCALE-STUDIES |
| PTGS2 | EDNRB | 0.0005 | Genetic Interactions | Lin-Smith-2010 |
| LIFR | VCAM1 | 0.0006 | Genetic Interactions | Lin-Smith-2010 |
| EDN3 | VCAM1 | 0.0008 | Genetic Interactions | Lin-Smith-2010 |
| EDN3 | LIFR | 0.0008 | Genetic Interactions | Lin-Smith-2010 |
| ITGA9 | LIFR | 0.0006 | Genetic Interactions | Lin-Smith-2010 |
| ITGA9 | EDN3 | 0.0007 | Genetic Interactions | Lin-Smith-2010 |
| EDN2 | ITGA9 | 0.0006 | Genetic Interactions | Lin-Smith-2010 |
| PTGIS | LIFR | 0.0005 | Genetic Interactions | Lin-Smith-2010 |
| PTGIS | EDN2 | 0.0005 | Genetic Interactions | Lin-Smith-2010 |
| TBXAS1 | EDN3 | 0.0012 | Genetic Interactions | Lin-Smith-2010 |
| TBXAS1 | PTGIS | 0.0008 | Genetic Interactions | Lin-Smith-2010 |
| TBXAS1 | SLC9A3 | 0.0016 | Genetic Interactions | Lin-Smith-2010 |
| GC | LIFR | 0.0006 | Genetic Interactions | Lin-Smith-2010 |
| GC | PTGIS | 0.0005 | Genetic Interactions | Lin-Smith-2010 |
| GC | PTGS1 | 0.0005 | Genetic Interactions | Lin-Smith-2010 |
| IL6ST | VCAM1 | 0.0008 | Genetic Interactions | Lin-Smith-2010 |
| IL6ST | EDN2 | 0.0007 | Genetic Interactions | Lin-Smith-2010 |
| IL6ST | GC | 0.0007 | Genetic Interactions | Lin-Smith-2010 |
| EDN1 | PTGIS | 0.0010 | Genetic Interactions | Lin-Smith-2010 |
| MYOCD | VCAM1 | 0.0015 | Genetic Interactions | Lin-Smith-2010 |
| MYOCD | SLC9A3 | 0.0024 | Genetic Interactions | Lin-Smith-2010 |
| LIFR | LIF | 0.1637 | Pathway | Wu-Stein-2010 |
| EDN3 | EDNRB | 0.1658 | Pathway | Wu-Stein-2010 |
| ITGA9 | VCAM1 | 0.0184 | Pathway | Wu-Stein-2010 |
| EDN2 | EDNRB | 0.1658 | Pathway | Wu-Stein-2010 |
| SLC9A3 | EDNRB | 0.3228 | Pathway | Wu-Stein-2010 |
| OSM | LIFR | 0.1612 | Pathway | Wu-Stein-2010 |
| ITGA4 | VCAM1 | 0.0157 | Pathway | Wu-Stein-2010 |
| IL6ST | LIF | 0.0660 | Pathway | Wu-Stein-2010 |
| IL6ST | LIFR | 0.0214 | Pathway | Wu-Stein-2010 |
| IL6ST | OSM | 0.0649 | Pathway | Wu-Stein-2010 |
| EDN1 | EDNRB | 0.0277 | Pathway | Wu-Stein-2010 |
| EDN1 | SLC9A3 | 0.1117 | Pathway | Wu-Stein-2010 |
| MYH11 | ACTA2 | 0.0543 | Pathway | Wu-Stein-2010 |
| MSN | VCAM1 | 0.1297 | Pathway | Wu-Stein-2010 |
| EDN3 | EDNRB | 0.4150 | Pathway | NCI_NATURE |
| ITGA9 | VCAM1 | 0.0500 | Pathway | NCI_NATURE |
| EDN2 | EDNRB | 0.3523 | Pathway | NCI_NATURE |
| SLC9A3 | EDNRB | 0.4263 | Pathway | NCI_NATURE |
| ITGA4 | VCAM1 | 0.0328 | Pathway | NCI_NATURE |
| EDN1 | EDNRB | 0.0702 | Pathway | NCI_NATURE |
| EDN1 | SLC9A3 | 0.1889 | Pathway | NCI_NATURE |
| MYOCD | ACTA2 | 0.3866 | Pathway | NCI_NATURE |
| ITGAD | VCAM1 | 0.3607 | Pathway | NCI_NATURE |
| EDN3 | EDNRB | 0.0100 | Physical Interactions | IREF-reactome |
| ITGA9 | VCAM1 | 0.4228 | Physical Interactions | IREF-reactome |
| EDN2 | EDNRB | 0.0100 | Physical Interactions | IREF-reactome |
| PTGIS | PTGS2 | 0.3103 | Physical Interactions | IREF-reactome |
| TBXAS1 | PTGS2 | 0.3103 | Physical Interactions | IREF-reactome |
| PTGDS | PTGS2 | 0.3103 | Physical Interactions | IREF-reactome |
| PTGS1 | PTGIS | 0.4225 | Physical Interactions | IREF-reactome |
| PTGS1 | TBXAS1 | 0.4225 | Physical Interactions | IREF-reactome |
| PTGS1 | PTGDS | 0.4225 | Physical Interactions | IREF-reactome |
| ITGA4 | VCAM1 | 0.2230 | Physical Interactions | IREF-reactome |
| EDN1 | EDNRB | 0.0100 | Physical Interactions | IREF-reactome |
| MYL12A | ACTA2 | 0.0485 | Physical Interactions | IREF-reactome |
| MYH11 | ACTA2 | 0.0524 | Physical Interactions | IREF-reactome |
| MYH11 | MYL12A | 0.0412 | Physical Interactions | IREF-reactome |
| EDN3 | EDNRB | 0.0100 | Physical Interactions | Vastrik-Stein-2007 |
| ITGA9 | VCAM1 | 0.4228 | Physical Interactions | Vastrik-Stein-2007 |
| EDN2 | EDNRB | 0.0100 | Physical Interactions | Vastrik-Stein-2007 |
| PTGIS | PTGS2 | 0.3103 | Physical Interactions | Vastrik-Stein-2007 |
| TBXAS1 | PTGS2 | 0.3103 | Physical Interactions | Vastrik-Stein-2007 |
| PTGDS | PTGS2 | 0.3103 | Physical Interactions | Vastrik-Stein-2007 |
| PTGS1 | PTGIS | 0.4225 | Physical Interactions | Vastrik-Stein-2007 |
| PTGS1 | TBXAS1 | 0.4225 | Physical Interactions | Vastrik-Stein-2007 |
| PTGS1 | PTGDS | 0.4225 | Physical Interactions | Vastrik-Stein-2007 |
| ITGA4 | VCAM1 | 0.2230 | Physical Interactions | Vastrik-Stein-2007 |
| EDN1 | EDNRB | 0.0100 | Physical Interactions | Vastrik-Stein-2007 |
| MYL12A | ACTA2 | 0.0485 | Physical Interactions | Vastrik-Stein-2007 |
| MYH11 | ACTA2 | 0.0524 | Physical Interactions | Vastrik-Stein-2007 |
| MYH11 | MYL12A | 0.0412 | Physical Interactions | Vastrik-Stein-2007 |
| LIFR | LIF | 0.5630 | Physical Interactions | IREF-quickgo |
| EDN3 | EDNRB | 0.5774 | Physical Interactions | IREF-quickgo |
| EDN2 | EDNRB | 0.5774 | Physical Interactions | IREF-quickgo |
| GC | ACTA2 | 1.0000 | Physical Interactions | IREF-quickgo |
| IL6ST | LIF | 0.2727 | Physical Interactions | IREF-quickgo |
| IL6ST | OSM | 0.2427 | Physical Interactions | IREF-quickgo |
| EDN1 | EDNRB | 0.5774 | Physical Interactions | IREF-quickgo |
| MSN | VCAM1 | 0.3251 | Physical Interactions | IREF-quickgo |
| LIFR | LIF | 0.6616 | Physical Interactions | IREF-dip |
| OSM | LIFR | 0.3055 | Physical Interactions | IREF-dip |
| IL6ST | OSM | 0.2152 | Physical Interactions | IREF-dip |
| MYL12A | ACTA2 | 0.5774 | Physical Interactions | IREF-mbinfo |
| MYH11 | MYL12A | 0.5774 | Physical Interactions | IREF-mbinfo |
| MYH11 | ACTA2 | 0.0816 | Physical Interactions | Havugimana-Emili-2012 |
| LIFR | LIF | 0.6369 | Physical Interactions | BIOGRID-SMALL-SCALE-STUDIES |
| EDN3 | EDNRB | 0.7860 | Physical Interactions | BIOGRID-SMALL-SCALE-STUDIES |
| PTGIS | PTGS2 | 0.1573 | Physical Interactions | BIOGRID-SMALL-SCALE-STUDIES |
| PTGS1 | PTGS2 | 0.1071 | Physical Interactions | BIOGRID-SMALL-SCALE-STUDIES |
| PTGS1 | PTGIS | 0.5514 | Physical Interactions | BIOGRID-SMALL-SCALE-STUDIES |
| OSM | LIFR | 0.3680 | Physical Interactions | BIOGRID-SMALL-SCALE-STUDIES |
| IL6ST | LIFR | 0.0932 | Physical Interactions | BIOGRID-SMALL-SCALE-STUDIES |
| ITGAD | VCAM1 | 0.1725 | Physical Interactions | BIOGRID-SMALL-SCALE-STUDIES |
| MSN | VCAM1 | 0.0421 | Physical Interactions | BIOGRID-SMALL-SCALE-STUDIES |
| ITGA4 | VCAM1 | 0.0344 | Physical Interactions | Humphries-Humphries-2009 |
| MSN | VCAM1 | 0.0344 | Physical Interactions | Humphries-Humphries-2009 |
| GC | ACTA2 | 0.0397 | Physical Interactions | Huttlin-Harper-2017 |
| IL6ST | OSM | 0.6539 | Physical Interactions | Huttlin-Harper-2017 |
| LIFR | LIF | 0.4362 | Physical Interactions | IREF-mint |
| OSM | LIFR | 0.3671 | Physical Interactions | IREF-mint |
| IL6ST | LIF | 0.3771 | Physical Interactions | IREF-mint |
| IL6ST | OSM | 0.3174 | Physical Interactions | IREF-mint |
| IL6ST | LIF | 0.5441 | Physical Interactions | IREF-bind-translation |
| IL6ST | LIF | 0.5564 | Physical Interactions | IREF-bind |
| IL6ST | OSM | 0.5474 | Physical Interactions | Huttlin-Gygi-2015 |
| LIFR | LIF | 0.5489 | Physical Interactions | IREF-matrixdb |
| OSM | LIFR | 0.2413 | Physical Interactions | IREF-matrixdb |
| IL6ST | LIF | 0.2395 | Physical Interactions | IREF-matrixdb |
| IL6ST | OSM | 0.1053 | Physical Interactions | IREF-matrixdb |
| EDN1 | EDNRB | 0.6025 | Physical Interactions | IREF-matrixdb |
| MYL12A | ACTA2 | 0.5498 | Physical Interactions | IREF-matrixdb |
| EDN1 | EDNRB | 0.4631 | Physical Interactions | Lim-Zoghbi-2006 |
| LIFR | LIF | 0.6616 | Physical Interactions | IREF-spike |
| OSM | LIFR | 0.4381 | Physical Interactions | IREF-spike |
| IL6ST | OSM | 0.3272 | Physical Interactions | IREF-spike |
| EDN1 | EDNRB | 0.2564 | Physical Interactions | IREF-spike |
| HTR1B | PTGS2 | 0.6418 | Physical Interactions | IREF-spike |
| LIFR | LIF | 0.6501 | Physical Interactions | IREF-biogrid |
| PTGIS | PTGS2 | 0.3386 | Physical Interactions | IREF-biogrid |
| OSM | LIFR | 0.1584 | Physical Interactions | IREF-biogrid |
| ITGA4 | VCAM1 | 0.0014 | Physical Interactions | IREF-biogrid |
| MYL12A | ITGA4 | 0.0036 | Physical Interactions | IREF-biogrid |
| ITGAD | VCAM1 | 0.0301 | Physical Interactions | IREF-biogrid |
| MSN | VCAM1 | 0.0034 | Physical Interactions | IREF-biogrid |
| MSN | ITGA4 | 0.0032 | Physical Interactions | IREF-biogrid |
| PTGS1 | PTGS2 | 1.0000 | Predicted | Wu-Stein-2010 |
| GC | ACTA2 | 0.1785 | Predicted | Wu-Stein-2010 |
| IL6ST | LIFR | 0.3735 | Predicted | I2D-BioGRID-Mouse2Human |
| EDN2 | EDN3 | 0.5000 | Shared protein domains | INTERPRO |
| TBXAS1 | PTGIS | 0.0128 | Shared protein domains | INTERPRO |
| PTGS1 | PTGS2 | 0.0896 | Shared protein domains | INTERPRO |
| OSM | LIF | 0.1053 | Shared protein domains | INTERPRO |
| ITGA4 | ITGA9 | 0.0617 | Shared protein domains | INTERPRO |
| IL6ST | LIFR | 0.0365 | Shared protein domains | INTERPRO |
| EDN1 | EDN3 | 0.5000 | Shared protein domains | INTERPRO |
| EDN1 | EDN2 | 0.5000 | Shared protein domains | INTERPRO |
| ITGAD | ITGA9 | 0.0410 | Shared protein domains | INTERPRO |
| ITGAD | ITGA4 | 0.0410 | Shared protein domains | INTERPRO |
| EDN2 | EDN3 | 0.5000 | Shared protein domains | PFAM |
| TBXAS1 | PTGIS | 0.0164 | Shared protein domains | PFAM |
| PTGS1 | PTGS2 | 0.0448 | Shared protein domains | PFAM |
| OSM | LIF | 1.0000 | Shared protein domains | PFAM |
| ITGA4 | ITGA9 | 0.0690 | Shared protein domains | PFAM |
| IL6ST | LIFR | 0.0144 | Shared protein domains | PFAM |
| EDN1 | EDN3 | 0.5000 | Shared protein domains | PFAM |
| EDN1 | EDN2 | 0.5000 | Shared protein domains | PFAM |
| HTR1B | EDNRB | 0.0035 | Shared protein domains | PFAM |
| ITGAD | ITGA9 | 0.0378 | Shared protein domains | PFAM |
| ITGAD | ITGA4 | 0.0378 | Shared protein domains | PFAM |

**Supplementary Table 3** | Data of TF-gene interactions.

| Id | Label | Degree | Betweenness |
| --- | --- | --- | --- |
| 3976 | LIF | 20 | 266 |
| 59 | ACTA2 | 4 | 66 |
| 51742 | ARID4B | 2 | 80 |
| 865 | CBFB | 1 | 0 |
| 56897 | WRNIP1 | 1 | 0 |
| 3726 | JUNB | 1 | 0 |
| 6670 | SP3 | 1 | 0 |
| 84905 | ZNF341 | 1 | 0 |
| 3090 | HIC1 | 1 | 0 |
| 10127 | ZNF263 | 1 | 0 |
| 4150 | MAZ | 1 | 0 |
| 84684 | INSM2 | 1 | 0 |
| 466 | ATF1 | 1 | 0 |
| 4089 | SMAD4 | 1 | 0 |
| 602 | BCL3 | 1 | 0 |
| 148979 | GLIS1 | 1 | 0 |
| 2738 | GLI4 | 1 | 0 |
| 121340 | SP7 | 1 | 0 |
| 53335 | BCL11A | 1 | 0 |
| 83463 | MXD3 | 1 | 0 |
| 7629 | ZNF76 | 1 | 0 |
| 1385 | CREB1 | 1 | 0 |
| 8187 | ZNF239 | 1 | 0 |
| 22890 | ZBTB1 | 1 | 0 |
| 84662 | GLIS2 | 1 | 0 |

**Supplementary Table 4** | Data of TF-miRNA co-regulatory network.

| Id | Label | Degree | Betweenness |
| --- | --- | --- | --- |
| 5743 | PTGS2 | 51 | 4638.5 |
| 7412 | VCAM1 | 38 | 3456.3 |
| 3976 | LIF | 30 | 2810.9 |
| 1910 | EDNRB | 19 | 1616.0 |
| 59 | ACTA2 | 13 | 1250.4 |
| 4790 | NFKB1 | 4 | 1150.5 |
| 10664 | CTCF | 3 | 607.6 |
| 2113 | ETS1 | 3 | 503.2 |
| MIMAT0000257 | hsa-miR-181b | 3 | 503.2 |
| MIMAT0002821 | hsa-miR-181d | 3 | 503.2 |
| 5970 | RELA | 3 | 460.4 |
| 4791 | NFKB2 | 3 | 460.4 |
| 6667 | SP1 | 2 | 335.2 |
| MIMAT0004692 | hsa-miR-340 | 2 | 251.0 |
| MIMAT0004801 | hsa-miR-590-3p | 2 | 251.0 |
| MIMAT0000082 | hsa-miR-26a | 2 | 175.5 |
| 3725 | JUN | 2 | 138.4 |
| 1051 | CEBPB | 2 | 138.4 |
| MIMAT0000256 | hsa-miR-181a | 2 | 138.4 |
| MIMAT0000086 | hsa-miR-29a | 2 | 96.9 |
| MIMAT0000100 | hsa-miR-29b | 2 | 96.9 |
| MIMAT0000681 | hsa-miR-29c | 2 | 96.9 |
| 7020 | TFAP2A | 1 | 0.0 |
| 7392 | USF2 | 1 | 0.0 |
| 7391 | USF1 | 1 | 0.0 |
| 7157 | TP53 | 1 | 0.0 |
| 7022 | TFAP2C | 1 | 0.0 |
| 6908 | TBP | 1 | 0.0 |
| 6773 | STAT2 | 1 | 0.0 |
| 6772 | STAT1 | 1 | 0.0 |
| 6722 | SRF | 1 | 0.0 |
| 6688 | SPI1 | 1 | 0.0 |
| 4089 | SMAD4 | 1 | 0.0 |
| 6239 | RREB1 | 1 | 0.0 |
| 5971 | RELB | 1 | 0.0 |
| 5966 | REL | 1 | 0.0 |
| 5915 | RARB | 1 | 0.0 |
| 5468 | PPARG | 1 | 0.0 |
| 5465 | PPARA | 1 | 0.0 |
| 5451 | POU2F1 | 1 | 0.0 |
| 5087 | PBX1 | 1 | 0.0 |
| 4782 | NFIC | 1 | 0.0 |
| 7593 | MZF1 | 1 | 0.0 |
| 4609 | MYC | 1 | 0.0 |
| 4602 | MYB | 1 | 0.0 |
| 2353 | FOS | 1 | 0.0 |
| 2118 | ETV4 | 1 | 0.0 |
| 2114 | ETS2 | 1 | 0.0 |
| 2002 | ELK1 | 1 | 0.0 |
| 1960 | EGR3 | 1 | 0.0 |
| 1874 | E2F4 | 1 | 0.0 |
| 1870 | E2F2 | 1 | 0.0 |
| 1385 | CREB1 | 1 | 0.0 |
| 1052 | CEBPD | 1 | 0.0 |
| 1050 | CEBPA | 1 | 0.0 |
| 468 | ATF4 | 1 | 0.0 |
| 1386 | ATF2 | 1 | 0.0 |
| 466 | ATF1 | 1 | 0.0 |
| 367 | AR | 1 | 0.0 |
| 1387 | CREBBP | 1 | 0.0 |
| 2033 | EP300 | 1 | 0.0 |
| 10725 | NFAT5 | 1 | 0.0 |
| 4772 | NFATC1 | 1 | 0.0 |
| 4773 | NFATC2 | 1 | 0.0 |
| 4775 | NFATC3 | 1 | 0.0 |
| 4776 | NFATC4 | 1 | 0.0 |
| MIMAT0000063 | hsa-let-7b | 1 | 0.0 |
| MIMAT0000099 | hsa-miR-101 | 1 | 0.0 |
| MIMAT0000422 | hsa-miR-124 | 1 | 0.0 |
| MIMAT0000443 | hsa-miR-125a-5p | 1 | 0.0 |
| MIMAT0000423 | hsa-miR-125b | 1 | 0.0 |
| MIMAT0000445 | hsa-miR-126 | 1 | 0.0 |
| MIMAT0005886 | hsa-miR-1297 | 1 | 0.0 |
| MIMAT0000426 | hsa-miR-132 | 1 | 0.0 |
| MIMAT0000429 | hsa-miR-137 | 1 | 0.0 |
| MIMAT0000434 | hsa-miR-142-3p | 1 | 0.0 |
| MIMAT0000435 | hsa-miR-143 | 1 | 0.0 |
| MIMAT0000436 | hsa-miR-144 | 1 | 0.0 |
| MIMAT0000437 | hsa-miR-145 | 1 | 0.0 |
| MIMAT0000439 | hsa-miR-153 | 1 | 0.0 |
| MIMAT0000069 | hsa-miR-16 | 1 | 0.0 |
| MIMAT0000258 | hsa-miR-181c | 1 | 0.0 |
| MIMAT0000231 | hsa-miR-199a | 1 | 0.0 |
| MIMAT0000682 | hsa-miR-200a | 1 | 0.0 |
| MIMAT0002811 | hsa-miR-202 | 1 | 0.0 |
| MIMAT0000076 | hsa-miR-21 | 1 | 0.0 |
| MIMAT0000271 | hsa-miR-214 | 1 | 0.0 |
| MIMAT0000081 | hsa-miR-25 | 1 | 0.0 |
| MIMAT0000083 | hsa-miR-26b | 1 | 0.0 |
| MIMAT0000084 | hsa-miR-27a | 1 | 0.0 |
| MIMAT0000419 | hsa-miR-27b | 1 | 0.0 |
| MIMAT0000085 | hsa-miR-28-5p | 1 | 0.0 |
| MIMAT0002890 | hsa-miR-299-5p | 1 | 0.0 |
| MIMAT0000090 | hsa-miR-32 | 1 | 0.0 |
| MIMAT0000752 | hsa-miR-328 | 1 | 0.0 |
| MIMAT0000760 | hsa-miR-331-3p | 1 | 0.0 |
| MIMAT0000773 | hsa-miR-346 | 1 | 0.0 |
| MIMAT0000707 | hsa-miR-363 | 1 | 0.0 |
| MIMAT0000719 | hsa-miR-367 | 1 | 0.0 |
| MIMAT0000720 | hsa-miR-376c | 1 | 0.0 |
| MIMAT0000730 | hsa-miR-377 | 1 | 0.0 |
| MIMAT0004748 | hsa-miR-423-5p | 1 | 0.0 |
| MIMAT0001341 | hsa-miR-424 | 1 | 0.0 |
| MIMAT0001532 | hsa-miR-448 | 1 | 0.0 |
| MIMAT0003161 | hsa-miR-493 | 1 | 0.0 |
| MIMAT0002816 | hsa-miR-494 | 1 | 0.0 |
| MIMAT0002817 | hsa-miR-495 | 1 | 0.0 |
| MIMAT0002875 | hsa-miR-504 | 1 | 0.0 |
| MIMAT0002878 | hsa-miR-506 | 1 | 0.0 |
| MIMAT0002879 | hsa-miR-507 | 1 | 0.0 |
| MIMAT0004771 | hsa-miR-516a-5p | 1 | 0.0 |
| MIMAT0002833 | hsa-miR-520a-5p | 1 | 0.0 |
| MIMAT0002830 | hsa-miR-520f | 1 | 0.0 |
| MIMAT0002850 | hsa-miR-524-3p | 1 | 0.0 |
| MIMAT0004954 | hsa-miR-543 | 1 | 0.0 |
| MIMAT0003165 | hsa-miR-545 | 1 | 0.0 |
| MIMAT0003285 | hsa-miR-548c-3p | 1 | 0.0 |
| MIMAT0003221 | hsa-miR-557 | 1 | 0.0 |
| MIMAT0003242 | hsa-miR-577 | 1 | 0.0 |
| MIMAT0003244 | hsa-miR-579 | 1 | 0.0 |
| MIMAT0003252 | hsa-miR-586 | 1 | 0.0 |
| MIMAT0003268 | hsa-miR-600 | 1 | 0.0 |
| MIMAT0003330 | hsa-miR-654-5p | 1 | 0.0 |
| MIMAT0003945 | hsa-miR-765 | 1 | 0.0 |
| MIMAT0003948 | hsa-miR-770-5p | 1 | 0.0 |
| MIMAT0004911 | hsa-miR-874 | 1 | 0.0 |
| MIMAT0000092 | hsa-miR-92a | 1 | 0.0 |
| MIMAT0003218 | hsa-miR-92b | 1 | 0.0 |
| MIMAT0000093 | hsa-miR-93 | 1 | 0.0 |
| MIMAT0004978 | hsa-miR-935 | 1 | 0.0 |
| MIMAT0004987 | hsa-miR-944 | 1 | 0.0 |
